# Long-range linkage disequilibrium in French beef cattle breeds

**DOI:** 10.1186/s12711-021-00657-8

**Published:** 2021-07-23

**Authors:** Abdelmajid El Hou, Dominique Rocha, Eric Venot, Véronique Blanquet, Romain Philippe

**Affiliations:** 1 INRAE, PEIRENE EA7500, USC1061 GAMAA, Université de Limoges, 87060 Limoges, France; 2 INRAE, AgroParisTech, GABI, Université Paris-Saclay, 78350 Jouy-en-Josas, France

## Abstract

**Background:**

Linkage disequilibrium (LD) is a key parameter to study the history of populations and to identify and fine map quantitative trait loci (QTL) and it has been studied for many years in animal populations. The advent of new genotyping technologies has allowed whole-genome LD studies in most cattle populations. However, to date, long-range LD (LRLD) between distant variants on the genome has not been investigated in detail in cattle. Here, we present the first comprehensive study of LRLD in French beef cattle by analysing data on 672 Charolais (CHA), 462 Limousine (LIM) and 326 Blonde d’Aquitaine (BLA) individuals that were genotyped on the Illumina BovineHD Beadchip. Furthermore, whole-genome LD and haplotype block structure were analysed in these three breeds.

**Results:**

We computed linkage disequilibrium (*r*^2^) values for 5.9, 5.6 and 6.0 billion pairs of SNPs on the 29 autosomes of CHA, LIM and BLA, respectively. Mean *r*^2^ values drop to less than 0.1 for distances between SNPs greater than 120 kb. However, for the first time, we detected the existence of LRLD in the three main French beef breeds. In total, 598, 266, and 795 LRLD events (*r*^2^ ≥ 0.6) were detected in CHA, LIM and BLA, respectively. Each breed had predominantly population-specific LRLD interactions, although shared LRLD events occurred in a number of regions (55 LRLD events were shared between two breeds and nine between the three breeds). Examples of possible functional gene interactions and QTL co-location were observed with some of these LRLD events, which suggests epistatic selection.

**Conclusions:**

We identified long-range linkage disequilibrium for the first time in French beef cattle populations. Epistatic selection may be the main source of the observed LRLD events, but other forces may also be involved. LRLD information should be accounted for in genome-wide association studies.

**Supplementary Information:**

The online version contains supplementary material available at 10.1186/s12711-021-00657-8.

## Background

Linkage disequilibrium (LD), or non-random association of alleles between loci, is important for identifying and fine mapping quantitative trait loci (QTL) [1]. The number of markers required for a successful association analysis and marker-assisted selection depends on the extent of LD across the genome. LD information also provides information on the history of populations. It is a useful alternative for estimating effective population size (Ne) when pedigree information is not available [2, 3]. Furthermore, LD information is used to detect recent positive selection (e.g. in humans [4] and *Drosophila* [5]). In addition, information about the extent and patterns of LD can provide important insights for the design of strategies to identify the genetic basis of complex phenotypes or to develop genomic selection methods [6].

The development of high-throughput genotyping technologies [7–9] and the availability of high-density (HD) single nucleotide polymorphism (SNP) panels have made it possible to carry out detailed studies on LD across the whole genome in cattle (e.g. [10, 11]). Different measures of LD have been published in the literature (e.g. [12]). The most frequently used measures in animal populations are the square correlation coefficient ($r^{2}$) [13] and the normalized *D*′ [14]. $r^{2}$ ranges from 0 (no-disequilibrium) to 1 (complete disequilibrium), and *D*′ from − 1 to 1. $r^{2}$ is the preferred measure of LD in animal populations, because it is less sensitive to population size than *D*′, and *D*′ tends to be inflated with small sample sizes and/or low allele frequencies [15, 16].

LD values decrease as the distance between markers on the genome increases. Most of the bovine studies using SNP data have shown that the average LD was close to zero for distances between markers greater than 500 kb. For example, using a low density of markers (2670 SNPs) but a very large number of animals from eight breeds, McKay et al. [17] found that for a distance between SNPs greater than 500 kb, the average LD was close to zero. Another study on 1546 Holstein–Friesian bulls that were genotyped for 15,036 SNPs showed that the mean $r^{2}$ values fall below 0.1 for distances between 200 and 500 kb [10]. However, in a study on 395 beef cattle animals genotyped on the BovineHD BeadChip, Mokry et al*.* [18] found that the average $r^{2}$ ranged from 0.05 to 0.07 at distances between 400 and 500 kb, but that, at these distances, the LD phase could persist to 0.66.

Recent studies have shown the existence of long-range LD (LRLD) between pairs of distant variants within the human genome. By analysing 60 Yoruba individuals from Nigeria obtained from the HapMap data, Koch et al*.* [19] showed the presence of LRLD between pairs of SNPs at distances greater than 250 kb among the 22 human autosomes. Park [20] showed the occurrence of specific LRLD interactions in African, European and East-Asian human populations from the 1000 Genomes Project. Another study on rainbow trout using a low-density SNP panel (31,788 SNPs) revealed LRLD for distances greater than 1 Mb, but the sub-family structure that exists in the population analysed explains this LRLD [21].

Several factors may be at the origin of LRLD events, such as population admixture [22], genetic drift or epistatic selection [23], recurrent bottlenecks [24] or chromosome structural variations (e.g. [25]). Genome assembly errors can also be the source of false observed LRLD events [19].

Numerous whole-genome LD studies based on medium-density (MD) or high-density (HD) SNP data have been performed in most dairy and beef cattle populations, and several have already shown the existence of LRLD in some bovine breeds (e.g. Beghain et al. [11]); however, to date, LRLD has not been investigated in detail in cattle. In these previous studies, the distribution of LRLD events along the cattle genome and the potential functional interactions between regions on LRLD have not been analyzed. In our study, we studied for the first time the extent of LRLD in three French beef cattle breeds and we explored the hypothesis that epistatic selection could explain the LRLD events by searching for functional interactions between genes in LRLD.

## Methods

### Animals and genotyping

For this study, since we did not perform any experiments on animals, no ethical approval was required. We used SNP genotyping data that were obtained from the GEMBAL (multi-breed genomics of beef and dairy cattle) research project [26–28] and a large population of 672 Charolais (CHA), 462 Limousine (LIM) and 326 Blonde d’Aquitaine (BLA) animals that were genotyped with the BovineHD BeadChip (Illumina Inc., San Diego, CA).

### SNP quality control

SNPs located on sex chromosomes or without an assigned position in the ARS-UCD1.2 genome assembly (GenBank assembly Accession GCA 002263795.2) were discarded, as well as SNPs and animals with a low call rate (< 2%). SNP quality control (QC) was carried out based on a minor allele frequency (MAF) lower than 0.05 and on Hardy–Weinberg equilibrium test (< 10e−6). Individuals that deviated by more than ± 3 standard deviations from the mean of the heterozygosity rate were removed from the analysis, as well as individuals with cryptic relatedness (pi-hat threshold > 0.125: third degree relatives), based on a subset of pruned SNPs, using the *PLINK* v1.9 software [29], as recommended by Marees et al*.* [30].

Principal component analysis (PCA) was performed to determine the family structure of each population because it can have a strong impact on the LD pattern. PCA on SNP genotypes was performed using the *snpgdsPCA* function of the *SNPRelate R* package [31] based on pruned SNPs which were in approximate LD. The genotype matrix (individuals * SNPs) was used to calculate a correlation matrix by individuals, and then the eigenvector of this matrix was calculated. These eigenvectors were used to describe the population structure. The PCA analysis was followed by a cluster analysis on a matrix of genome-wide identity by state (IBS) pairwise distances using the *snpgdsHCluster* and *snpgdsIBS* functions of the *SNPRelate* package. Groups were determined by a permutation score using the same *R* package (*SNPRelate*).

### Linkage disequilibrium analysis

For each pair of SNPs, we calculated the square correlation coefficient ($r^{2}$) as a measure of LD using the *PLINK* v1.9 software [29] and for all syntenic pairs of SNPs on each autosome, we also calculated the $r^{2}$ between two loci [13]. Background LD measured as the $r^{2}$ between non-syntenic SNPs was estimated among a subset of non-syntenic pairs of SNPs, which were selected using the *-indep-pairwise* option of the *PLINK* software with the default parameters: 50 SNPs per window, a shift of five SNPs between windows at each step, and a pairwise $r^{2}$ threshold of 0.2.

### Haplotype block analysis

Haplotype block patterns were estimated using the *–blocks* option based on the *PLINK* v1.9 software [29]. The same QC filters were applied to the data of the three breeds. *PLINK* v1.9 uses the haplotype block definition suggested by Gabriel et al*.* [32], by default, with blocks of a maximum size of 5000 kb.

### Identification of long-range linkage disequilibrium

We defined long-range LD (henceforth LRLD event) between two haplotype blocks as follows: (1) at least two markers per haplotype block with an average $r^{2}$ higher than 0.4, 0.6 or 0.8; and (2) a haplotype block distance longer than or equal to 1 Mb on the same chromosome. We used the *Circos* software v0.69-6 [33] to visualize whole-genome LRLD events. By analysing the variance (one-way ANOVA), we checked whether the number of LRLD events was on average statistically different in the three breeds, and we used Spearman’s test to check the correlation between the number of LRLD events and chromosome size.

### LRLD and functional interactions

To check for the existence of functional interactions in the identified LRLD paired blocks, all the genes with in each block were retrieved from the Ensembl database (release 101) [34]. The Search Tool for the Retrieval of Interacting Genes (STRING) database [35] was queried for possible functional interactions between proteins encoded by genes within LRLD events. In addition, we used the cattle quantitative trait locus (QTL) database (Cattle QTLdb) [36] to investigate the presence of QTL associated with the same phenotype in both blocks, for each LRLD event. All 425 phenotypes of the Cattle QTLdb, including the 266 phenotypes related to meat/carcass and production traits were used. A common LRLD event between breeds was defined as the strict intersection of these LRLD events in each pair of breeds or in all three breeds. We checked if the proportion of LRLD paired blocks showing functional interactions (STRING interaction and QTL) was not simply due to sampling. Then, we used the Chi^2^ test between the proportion of LRLD events with STRING interactions or QTL and the same number of randomly chosen pairs of blocks.

## Results

### Quality control for LD analysis

To evaluate the extent of LD in the three main French beef cattle breeds, 672 CHA, 462 LIM and 326 BLA animals were genotyped using the BovineHD BeadChip. However, to eliminate bias in LD related to family structure, only the least related (third-degree relatives) animals were selected based on the pi-hat values. In total, 145 CHA, 106 LIM and 49 BLA animals passed QC, yielding 559,260, 541,319, and 563,740 SNPs for the CHA, LIM, and BLA animals, respectively.

These SNPs cover a total length of 2.48 Gb on the genome (Table 1) and (see Additional file 1: Tables S1–S3). SNPs were generally homogeneously distributed along the 29 autosomes, with fluctuations in some chromosomal regions (see Additional file 2: Figures S1–S3). Adjacent SNPs were separated by an average distance of 4.4 ± 7.0 kb, 4.6 ± 7.3 kb, 4.4 ± 6.9 kb for CHA, LIM and BLA, respectively and a median spacing of ~ 2.6 kb. All adjacent SNPs had an inter-distance shorter than 1 Mb, except *Bos taurus* (BTA) chromosomes 8 and 10 on which some adjacent SNPs had an inter-distance longer than 1 Mb.

**Table 1:** Distribution of SNPs along the autosomes of the Charolaise, Limousine and Blonde d’Aquitaine breeds

Breed	Number of SNPs	Size (Gb)	Average inter-distance (± sd) in kb	Median	Max (kb)
Charolaise	559,260	2.48	4.4 ± 6.9	2.6	1636.2
Limousine	541,319	2.48	4.6 ± 7.1	2.7	1636.7
Blonde d’Aquitaine	563,740	2.48	4.4 ± 6.7	2.6	1636.2

Principal component analysis in the three breeds showed that each population was homogeneous. These results were confirmed by a clustering method using a matrix of genome-wide identity by state (IBS) pairwise distances (see Additional file 3: Figures S4 and S5).

### Genome-wide linkage disequilibrium analysis

**Fig. 1:**
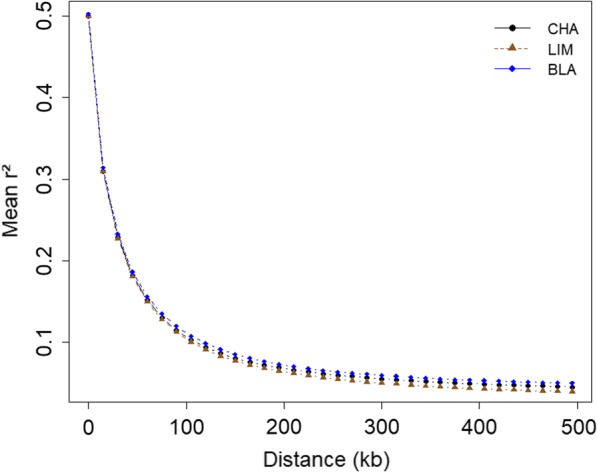
Distribution of average $r^{2}$ values for CHA, LIM and BLA breeds with respect to physical distance (kb) in 500-kb windows

We analysed the LD decay for SNPs within 500-kb windows. The mean values of $r^{2}$, pooled over autosomes for an inter-SNP distance of 15 kb are summarized in Additional file 4: Tables S4–S6. The distribution of the $r^{2}$ values according to the physical distance between loci is shown in Fig. 1. As expected, there was an inversely proportional relationship between the mean $r^{2}$ and the physical distance between SNPs in the three breeds (Fig. 1 and see Additional file 4: Tables S4–S6). The average $r^{2}$ values dropped below 0.1 at a distance greater than 120 kb. Background LD was estimated on a set of 50,083, 51,301, and 46,925 non-syntenic SNPs for CHA, LIM and BLA, respectively, and resulted in values of 0.009 for CHA, 0.010 for LIM and 0.024 for BLA.

The average values of LD ($r^{2}$) varied between 0.5 at distances smaller than 15 kb, to less than 0.1 at distances greater than 120 kb (Fig. 1 and see Additional file 4: Tables S4–S6). The mean $r^{2}$ (± SD) values between pairs of SNPs ranged from 0.079 (± 0.154) to 0.121 (± 0.203) for CHA, LIM and BLA (see Additional file 5: Tables S7–S9). In contrast to the LIM and BLA breeds, BTA14 of the CHA breed showed a lower LD decay with physical distance than the other chromosomes (see Additional file 6: Figures S6–S8).

### Haplotype block structure

We used the method defined by Gabriel et al. [32] based on genotyping data to identify the haplotype blocks on each autosome; the haplotype blocks that included only two SNPs (16,624 for CHA, 16,131 for LIM and 14,763 for BLA) were discarded to avoid formation of spurious blocks. In total, 460,224, 433,950 and 422,469 SNPs were clustered into haplotype blocks, which represent 82.29, 80.17, and 74.94% of all the SNPs for CHA (see Additional file 7: Table S10), LIM (see Additional file 7: Tables S11) and BLA (see Additional file 7: Tables S12), respectively. These haplotype blocks covered 1.55, 1.49, and 1.35 Gb of the total genome size for CHA, LIM and BLA, respectively (Table 2), and the chromosome coverage ranged from 45.87% (BTA23, BLA) to 67.42% (BTA7, CHA) in the three breeds (see Additional file 7: Tables S10–S12).

**Table 2:** Descriptive summary of the haplotype block analysis in the Charolais, Limousine and Blonde d’Aquitaine breeds

Breed	Number of SNPs	Chr size (Mb)	Number of blocks	Block coverage length (Mb)	Block coverage length (%)	Number of SNPs in blocks	% SNPs in blocks
Charolaise	559,260	2480.92	52,664	1551.78	62.55	460,224	82.29
Limousine	541,319	2481.03	50,553	1488.29	59.99	433,950	80.17
Blonde d’Aquitaine	563,740	2481.00	48,303	1352.07	54.50	422,469	74.94

Total number of SNPs, total chromosome (chr) size, number of blocks per breed, total length covered by haplotype blocks, block coverage length in percent, number of SNPs in blocks and percent of SNPs in blocks (% SNPs in blocks)

In the three breeds, we observed larger haplotypes on BTA6, 7, 12 and 23 than on the other chromosomes and we found small or no haplotype blocks on BTA10, 12 and 23 because of the low density of SNPs for these chomosomes (see Additional file 8: Figure S9 and Additional file 11: Table S13) for CHA, (see Additional file 9: Figure S10 and Additional file 11: Table S14) for LIM, and (see Additional file 10: Figure S11 and Additional file 11: Table S15) for BLA. In addition, large haplotype blocks were found at the extreme ends of BTA15, 21 and 23 in the three breeds.

**Fig. 2:**
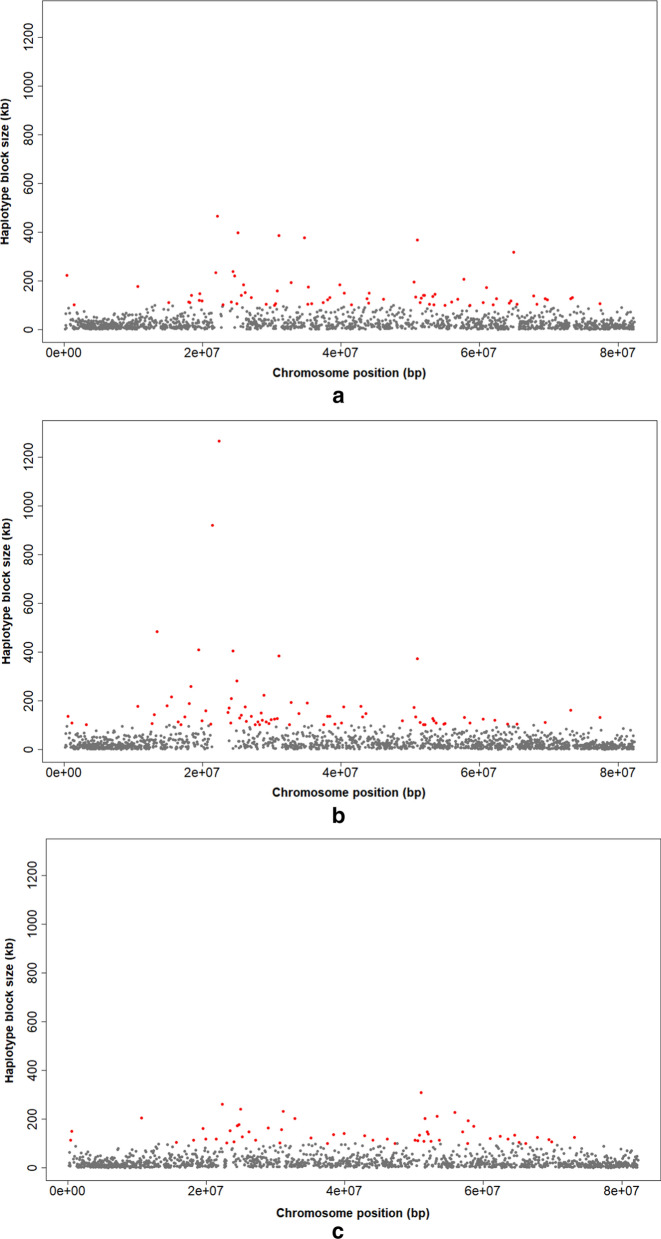
Size and distribution of haplotype blocks on chromosome 14 of CHA (**a**), LIM (**b**) and BLA (**c**). Red points: block size ≥ 100 kb

**Fig. 3:**
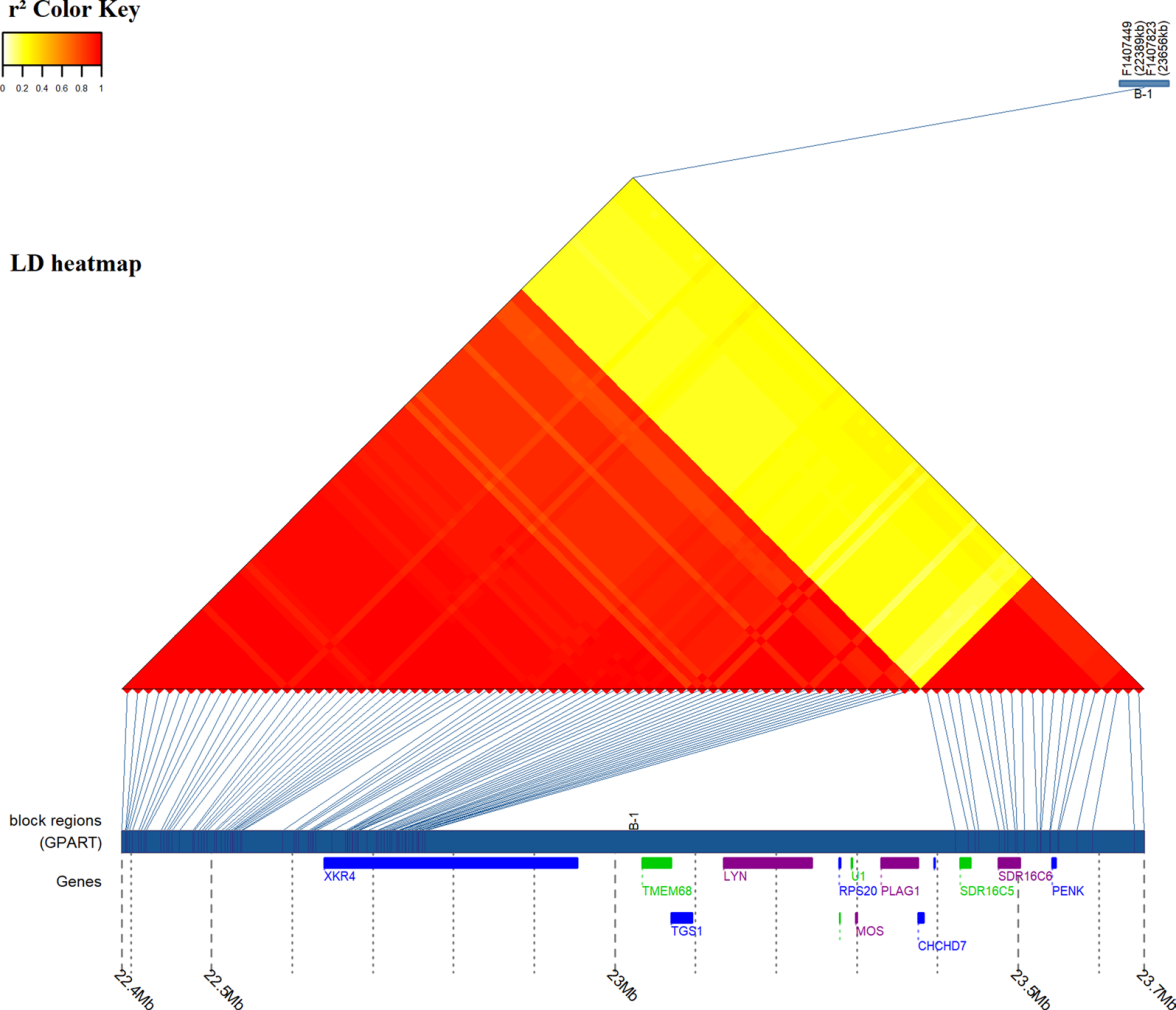
Chromosome 14 LD block heatmap of the region containing *PLAG1*. The region corresponds to the largest haplotype on BTA14 of CHA with annotated genes from Ensembl database

Interestingly, large haplotype blocks on BTA14 (size range from 800 to 1267 kb) were found for CHA, although block sizes did not exceed 500 kb in the other two breeds (Fig. 2), which suggests a selection pressure on this chromosome. The largest block on BTA14 hosts 12 annotated genes (*XKR4*, *TMEM68*, *TGS1*, *LYN*, *RP620*, *U1*, *MOS*, *PLAG1*, *CHCHD7*, *SDR16C5*, *SDR16C6*, and *PENK*), with *PLAG1* known to be associated with stature and carcass yield in cattle (Fig. 3) [37].

### Long-range linkage disequilibrium

**Fig. 4:**
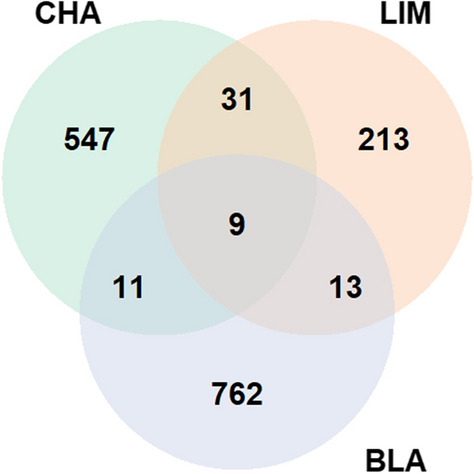
Number of shared LRLD regions between CHA, LIM and BLA breeds. CHA and LIM share 30 LRLD, CHA and BLA share 10 LRLD, LIM and BLA share 13 LRLD, and the three breeds share nine LRLD regions

In order to investigate the existence of LRLD in the three populations, the ($r^{2}$) measures of LD were calculated for each pair-wise combination of SNPs on each autosome (syntenic markers). In total, 5.9, 5.6, and 6.0 billion pairs were analysed for all the autosomes in the CHA, LIM and BLA breeds, respectively. In this study, we called LRLD between two haplotype blocks, if at least two SNPs in each block were in high LD ($r^{2}$ ≥ 0.4, 0.6 or 0.8) with SNPs in the other block, and if the two blocks had an inter-distance greater than or equal to 1 Mb. In total, 15, 266 and 3288 LRLD events were found for $r^{2}$ ≥ 0.8, 0.6 or 0.4, respectively, in LIM (see Additional file 12 Table S16), 29, 598 and 7840 LRLD events were found for $r^{2}$ ≥ 0.8, 0.6 or 0.4, respectively, in CHA (see Additional file 12 Table S17), and 61, 795 and 22,517 LRLD events were found for $r^{2}$ ≥ 0.8, 0.6 or 0.4, respectively in BLA (see Additional file 12 Table S18). A small number of LRLD events was observed for $r^{2}$ ≥ 0.8 and a very large number for $r^{2}$ ≥ 0.4 in the three breeds. In order to analyse a reasonable number of LRLD events, only LRLD events with a $r^{2}$ ≥ 0.6 will be described in the next section. Table 3 shows the number of LRLD events per breed ($r^{2}$ ≥ 0.6). In total, 598, 266 and 795 LRLD events were found for CHA, LIM and BLA, respectively, which indicates that LRLD occurs in these three beef cattle breeds. Each chromosome displayed a different number of LRLD events, with some chromosomes having few or no LRLD events depending on the population. The number of LRLD events in BLA was larger than in the other two breeds (*ANOVA*, p = 0.001) and the number of LRLD events was moderately correlated with chromosome size (*rho* = 0.58, p = 0.0010; *rho* = 0.49, p = 0.0064; *rho* = 0.7300, p = 8.6e−06 for CHA, LIM and BLA, respectively). The three breeds shared nine common LRLD events, CHA shared 31 LRLD events with LIM only and 11 with BLA only, and LIM shared 13 LRLD events with BLA only (Fig. 4 and see Additional file 13 Table S19).

**Table 3:** Descriptive summary of the LRLD events in the Charolaise, Limousine and Blonde d’Aquitaine breeds

BTA	Charolaise				Limousine				Blonde d’Aquitaine			
	N LRLD	Avg dist (Mb)	SD (Mb)	Max dist (Mb)	N LRLD	Avg dist (Mb)	SD (Mb)	Max dist (Mb)	N LRLD	Avg dist (Mb)	SD (Mb)	Max dist (Mb)
1	30	1.88	0.83	3.73	27	1.99	0.73	3.87	77	3.77	4.85	34.27
2	22	1.82	0.85	4.97	34	1.99	0.77	5.29	134	3.92	3.84	28.20
3	17	1.67	0.57	2.54	2	1.32	0.45	1.64	18	6.00	7.03	25.41
4	22	1.61	0.60	3.49	4	1.90	0.51	2.63	21	3.63	4.10	15.65
5	43	2.00	0.75	3.91	12	1.42	0.58	3.11	40	2.88	4.62	29.18
6	60	1.79	0.58	3.18	16	2.04	0.87	4.22	48	5.66	8.49	52.45
7	74	2.54	1.11	4.39	13	2.47	1.28	4.29	35	3.13	3.75	20.11
8	57	1.43	0.37	2.86	1	1.07	–	1.07	51	2.58	1.51	7.76
9	45	1.80	0.73	4.19	15	2.39	1.31	4.37	25	3.10	2.97	14.59
10	9	1.60	0.47	2.36	12	2.13	0.89	3.74	12	2.99	2.09	8.01
11	12	1.55	0.45	2.42	21	3.36	1.70	5.58	44	3.27	2.99	12.22
12	17	1.69	0.51	3.19	3	1.15	0.12	1.24	28	2.91	1.66	6.56
13	5	1.56	0.20	1.83	0	–	–	–	29	4.63	4.01	22.00
14	42	1.73	0.84	5.19	4	1.70	0.69	2.51	36	3.87	3.31	14.86
15	61	1.77	0.75	3.60	46	2.27	1.37	6.64	34	3.95	2.75	10.45
16	8	1.30	0.21	1.67	4	1.37	0.44	2.03	17	4.27	5.89	25.23
17	5	1.67	0.34	1.99	8	1.45	0.33	1.96	35	6.24	11.10	36.61
18	1	2.30	-	2.30	3	1.23	0.18	1.36	15	3.84	3.82	12.57
19	13	1.37	0.35	2.27	4	2.84	0.14	3.01	0	–	–	–
20	10	1.33	0.28	1.97	2	1.65	0.63	2.09	5	2.18	1.03	3.65
21	3	1.23	0.17	1.40	6	1.71	0.58	2.42	20	3.69	3.42	12.87
22	1	1.62	–	1.62	0	–	–	–	2	2.55	1.04	3.29
23	6	3.33	0.42	4.08	13	1.61	0.44	2.31	20	1.80	1.15	5.04
24	11	1.73	0.64	2.91	2	1.34	0.33	1.57	30	3.69	1.57	6.41
25	0	–	–	–	1	1.07	–	1.07	2	4.84	5.21	8.53
26	12	9.16	11.72	25.09	11	3.53	7.12	24.97	9	9.25	11.61	24.74
27	5	1.06	0.07	1.19	2	1.57	0.31	1.79	3	1.30	0.12	1.42
28	1	1.11	–	1.11	0	–	–	–	4	14.05	7.41	23.13
29	6	1.49	0.36	1.99	0	–	–	–	1	6.73	–	6.73
Total	598	1.97	2.05	25.09	266	2.15	1.79	24.97	795	3.92	4.96	52.45

N LRLD: number of LRLD events, Avg dist: average distance in Mb; SD: standard deviation; Max dist: distance: maximal distance between LRLD block pairs (in Mb)

**Fig. 5:**
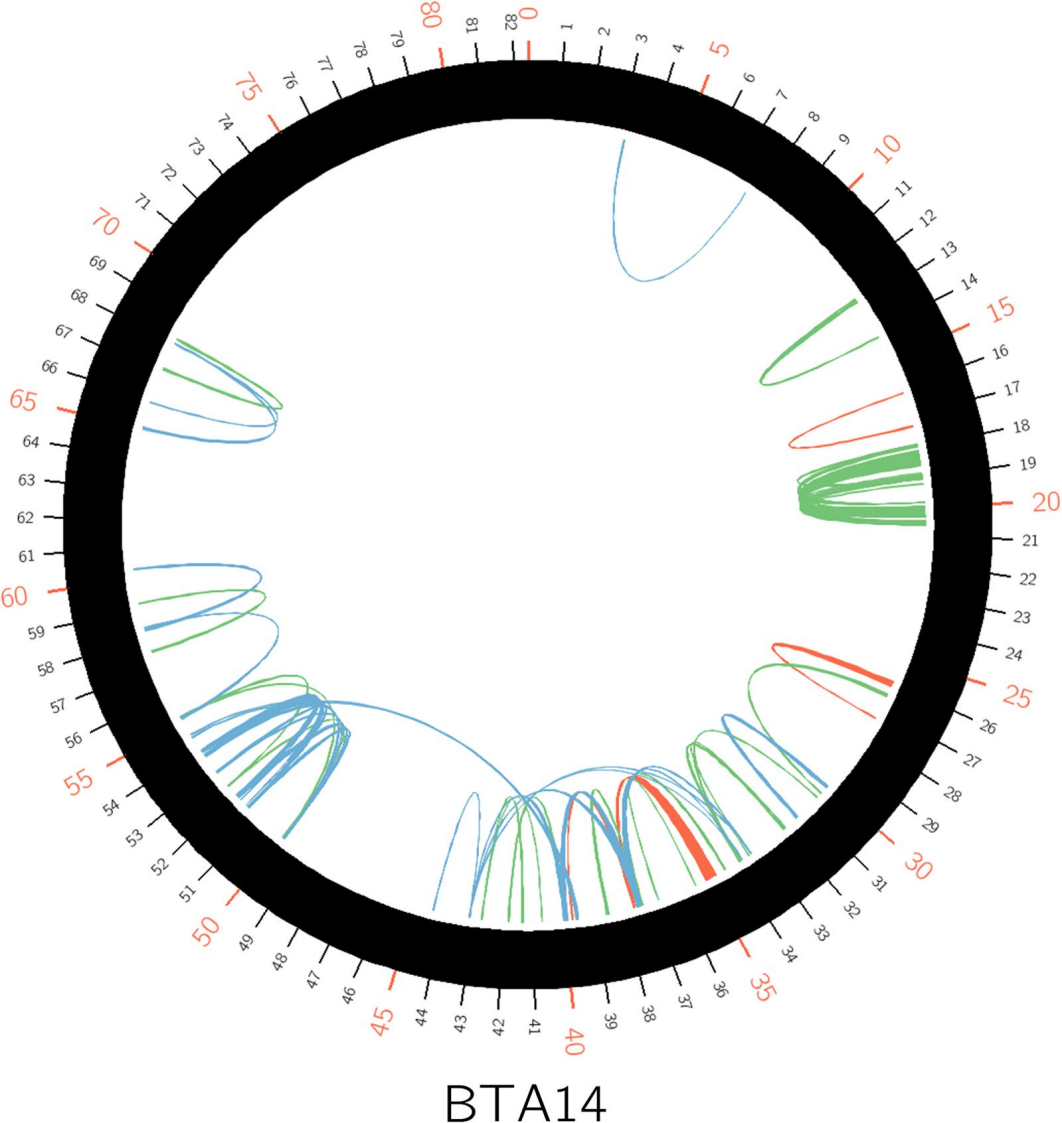
Chromosome-wide LRLD on BTA14 for each of the three breeds. Green for CHA, Red for LIM and Blue for BLA. Plots were obtained by using the *Circos* software

**Fig. 6:**
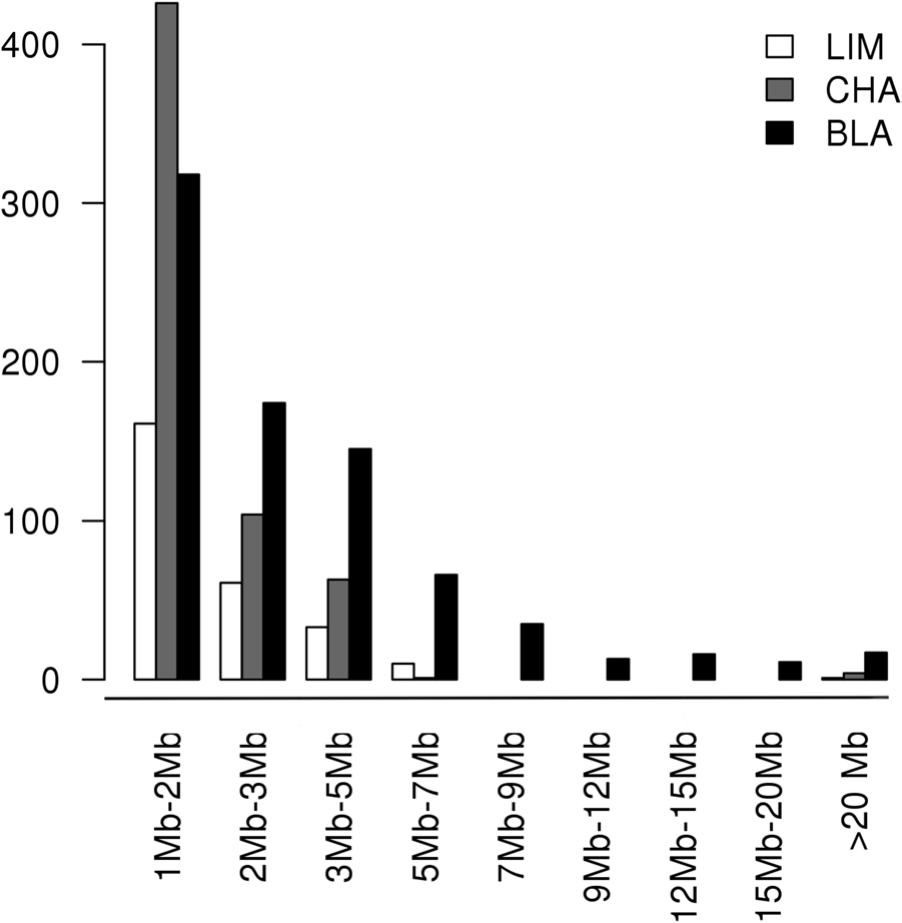
Number of LRLD events according to the ranges of haplotype block inter-distances in the three breeds. Intervals of the X-axis are not equal to one another

We looked at the specificity of chromosome-wide LRLD events in the three breeds (Fig. 5 and Additional file 14: Figure S12). Each breed had predominantly population-specific LRLD events, although common LRLD events also existed in a number of regions, as mentioned above. We were interested in the distance between pairs of LRLD blocks in each breed. The average distance between pairs of LRLD blocks was 1.97 ± 2.05 and 2.15 ± 1.79 Mb, for CHA and LIM, respectively (Table 3). However, this average distance on the whole genome was greater in the BLA breed (3.92 ± 4.96 Mb) and was the largest on almost all the chromosomes of the BLA breed. Interestingly, in BLA, 60% of the LRLD events were separated by more than 2 Mb, whereas in CHA and LIM only 28.76 and 39.47% were separated by more than 2 Mb, respectively (Fig. 6).

### LRLD functional interactions

**Fig. 7:**
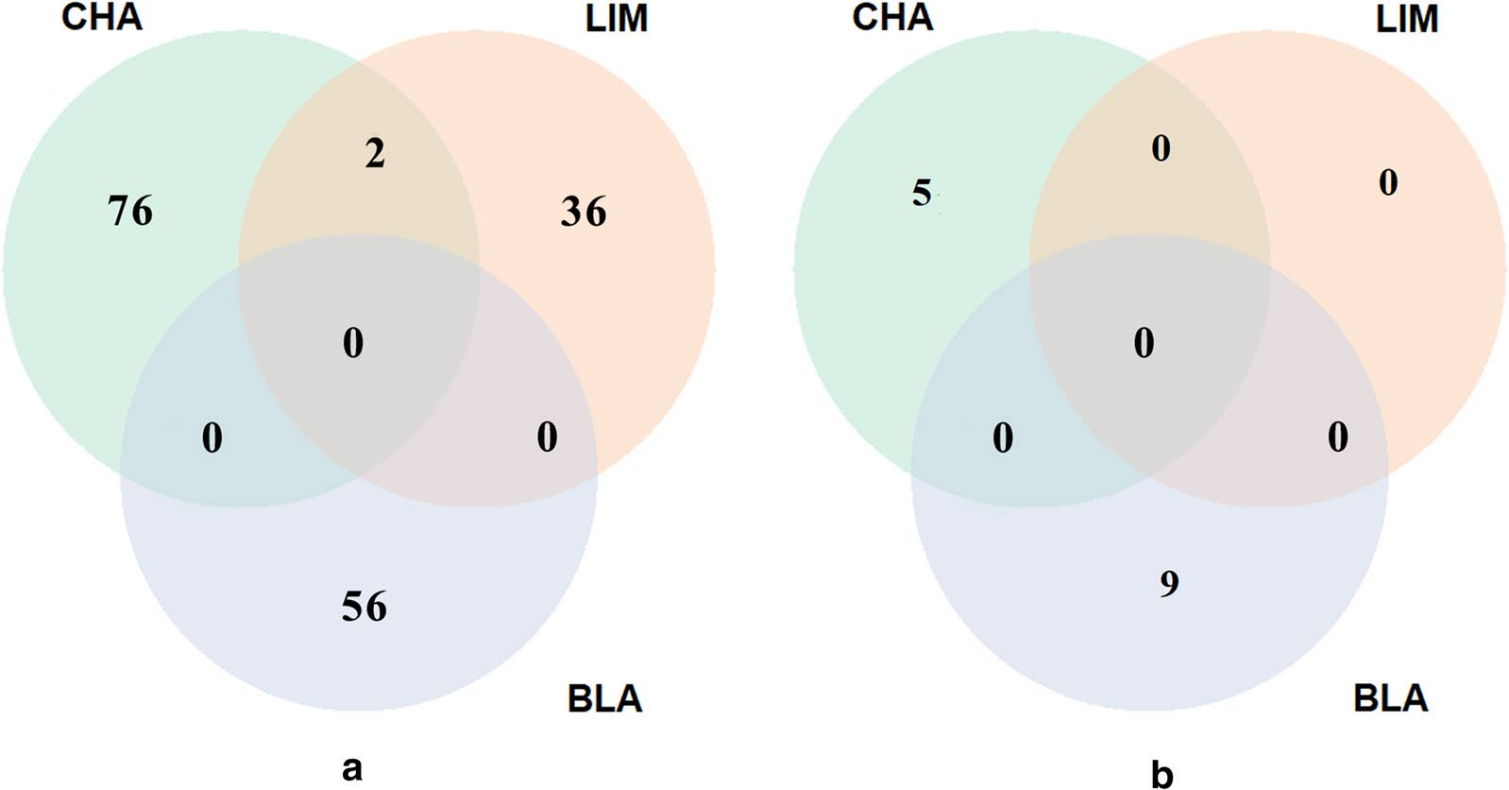
Summary of the number of LRLD events showing STRING (**a**) or QTL interactions (**b**) in the three breeds

Table 4 summarizes the number of LRLD events for which annotated genes were identified in both blocks and Fig. 7 shows the results of possible functional interactions (see Additional file 15: Tables S20–S22). Among the 598, 266 and 795 LRLD events in CHA, LIM and BLA, 270, 116 and 290 have annotated genes in both blocks, respectively. Fifteen of the shared LRLD events between two breeds have annotated genes in each of the blocks and no annotated genes were found in the nine regions shared between the three breeds.

**Table 4:** Number of LRLD events with annotated genes in both blocks

Breed	Number of LRLD events with genes^a^
Charolaise	270
Limousine	116
Blonde d’Aquitaine	290
Common (2 breeds)	15
Common (3 breeds)	0

^a^Number of LRLD events with annotated genes in both blocks

The STRING database was queried to see if, within each LRLD event, there were any potential interactions between the proteins encoded by the genes present in their two blocks. Such interactions were found for 78, 38, and 56 LRLD events in CHA, LIM and BLA, respectively, but for only two LRLD events that were shared between two breeds (Table 5 and Fig. 7a). Furthermore, no common LRLD events were identified between the three breeds with STRING interactions (Fig. 7a and see Additional file 15: Tables S20–S22). In order to investigate if these numbers of interactions were due to chance only, the same numbers of pairs of blocks were randomly selected in the three breeds i.e. 598 in CHA, 266 in LIM and 795 in BLA. In total, 16, 10 and 19 random pairs of blocks showed interactions in CHA, LIM and BLA, respectively. The Chi2 test between the random pairs of blocks and the LRLD events resulted in a p-value ranging from 1.204e−05 to 2.701e−11 (Table 5), which indicates that the interactions identified in the LRLD events were not due to sampling. Interestingly, we detected several examples of STRING-type text-mining interactions. For example, dentin matrix acidic phosphoprotein 1 encoded by *DMP1*, an extracellular protein involved in mineralization of dentin regulates dentin sialophosphoprotein encoded by *DSPP* [38]. The two genes, *DMP1* and *DSPP*, are located in two different blocks of the LRLD events 187–191 of in CHA (see Additional file 15: Table S20). Other examples are the *OR52W1* and *OR52B2* genes related to the olfactory system and the *CNGA4* (*cyclic nucleotide gated channel alpha 4*) gene, which are co-mentioned in PubMed Abstracts (text-mining) [39] and are in two different blocks of LRLD events 479–480 in CHA (see Additional file 15: Tables S20).

**Table 5:** Number of LRLD events and randomly-chosen pairs of blocks with functional interactions

Breed	Type of pairs of blocks	Number of pairs of blocks	Number of pairs of blocks with STRING interaction	P-value^a^
Charolaise	LRLD	598	78	2.701e−11
	Random	598	16	
Blonde d’Aquitaine	LRLD	795	56	1.204e−05
	Random	795	19	
Limousine	LRLD	266	38	2.668e−05
	Random	266	10	

^a^P-value of a Chi^2^ test between random pairs and LRLD pairs

The cattle QTL database was used to check for the potential existence of QTL associated with the same phenotype in the two blocks of each LRLD event that could be an indicator of epistasic interactions. In CHA, LIM and BLA respectively, 38, 30 and seven LRLD events with QTL associated with the same phenotype (in each block) were found (Table 6). We checked for traits that are related to selection objectives in beef cattle (i.e. QTL associated with production and carcass or meat quality traits). We did not identify LRLD events with related QTL in LIM, but we found five and nine LRLD events with QTL of interest in CHA and BLA, respectively (Fig. 7b and see Additional file 15: Tables S20, S21 and S22). In CHA, four of the LRLD events may be associated with body weight and one with shear force. In BLA, five of the LRLD events may be associated with body weight, two with scrotal circumference, one with average daily gain and one with muscle anserine content. In the same way as for STRING interactions, the impact of sampling was tested (Table 6). Even if only two of the p-values of the Chi2 test between the random pairs of blocks and the LRLD events were significant (the other non-significant p-values were probably due to the small sample sizes), we observed a clear trend that more QTL were associated with beef-related traits in the LRLD events than with the randomly-chosen pairs of blocks.

**Table 6:** Number of LRLD events and random pairs of blocks with potential QTL interactions

Breed	Type of pairs of blocks	Number of pairs of blocks	Number of pairs of blocks with all QTL^a^	P-value^b^	Number of pairs of blocks with production-related QTL^c^	P-value^b^
Charolaise	LRLD	598	38	1.704e−09	5	0.101
	Random	598	1		1	
Blonde d’Aquitaine	LRLD	795	30	1.439e−09	9	0.002
	random	795	1		0	
Limousine	LRLD	266	7	0.032	0	NA
	Random	266	1		0	

^a^Number of pairs of blocks with QTL for the same phenotype in the two blocks (all QTL of the Cattle QTLdb)

^b^P-value of a Chi^2^ test between random pairs and LRLD pairs

^c^Number of pairs of blocks with QTL for the same phenotype in the two blocks (only QTL for production, carcass and meat quality traits

## Discussion

In this study, we investigated the existence of LRLD events in the three main French beef cattle breeds for the first time. The study covered a relatively large population of genotyped animals, with a very high density of markers. Each population had a different number of LRLD events, which indicates that some long-range interactions are specific to each breed. Nonetheless, common LRLD regions also exist between the three breeds.

The average number of LRLD events was larger in BLA than in the other two breeds and the inter-distance between LRLD events reached up to ~ 52 Mb (BTA6) in BLA, but was not greater than 25.09 Mb in CHA and LIM (Table 3). There are several possible explanations for such LRLD in the BLA breed, including population admixture. Indeed, the BLA breed was formed by merging three French South-West Blonde populations (Quercy, Garonnais and Blonde des Pyrénées) in the 1960s [11]. However, PCA and clustering analyses have shown that each of these three populations was genetically homogeneous. The occurrence of bottlenecks can also explain this long range LD in the three breeds. Bouquet et al. [40] showed that the BLA population experienced a bottleneck since the 1970s due to the extensive use of artificial insemination by French breeders, which resulted in a decrease in effective population size (estimated at 247), compared to CHA (601) and LIM (> 1000). Bottlenecks have also been suggested as a source of large LD in the human genome [41]. In contrast, the unequal number of LRLD events per chromosome indicates that sub-populations and bottlenecks may not be the main source of the LRLD observed in this study, which suggests a role of selection in these populations.

Interestingly, in our study, we identified several functional interactions between genes from pairs of LRLD regions, which suggests epistatic selection. The functional interaction between genes in LRLD regions may explain some of the LD observed at long distances. Indeed, epistasis can contribute to LD between variants located at a long distance from each other on the same chromosome (e.g. [42]). For example, in CHA, SNPs in the *sin3A-associated protein 130 kDa* (*SAP130*) and *UDP-glucose glycoprotein glucosyltransferase 1* (*UGGT1*) genes were in LRLD, see block number 31 in (Additional file 15: Table S20). These genes are known to be involved in the acetylation of the H3 histone and in protein metabolism [43], respectively. A genome-wide association study (GWAS) has shown that SNPs located 500 kb upstream of these two genes are associated with average daily gain phenotype [44]. The *MYL6B* and *MYL6* genes in CHA, see block number108 in Additional file 15 Table S20 were identified in the Brown Hanwoo breed (Korean beef cattle) within a region under recent positive selection [45]. This region contains a QTL associated to a skeletal muscle generation phenotype [46]. Furthermore, the *CATSPER3* and *PITX1* genes in CHA, see block number 254 in Additional file 15 Table S20 were found in a candidate region under selection in European and African *Bos taurus* breeds [47]. In BLA, the *SNUPN*, *CSPG4*, and *PTPN9* genes, see block number 712 in Additional file 15 Table S22 were co-mentioned in PubMed Abstracts [48]. They have been identified in Korean cattle in selective sweep regions that are associated with marbling score [48]. In addition, the *OR52W1* and *OR52B2* genes related to the olfactory system, and the *cyclic nucleotide gated channel alpha 4* (*CNGA4*) gene were co-mentioned in PubMed Abstracts because they are involved in the olfactory transduction signal [39]. The *OR52W1* and *CNGA4* genes were identified in regions under selection in the Western Pyrénées sheep [39]. These genes are located in an LRLD event shared between CHA and LIM, see number 18 in Additional file 15: Tables S20 and S22. The orthologous genes of *FOXP2* and *MSANTD1* in CHA, see block number 82 in Additional file 15: Table S20, have been described as under selection in Moroccan Black and Northern goats, respectively [49]. The *DMP1* gene in CHA, see block number 187 in Additional file 15: Table S20, encodes an extracellular protein involved in the mineralization of dentin that regulates DSPP during dentinogenesis [38]. Interestingly, the two genes, *DMP1* and *DSPP*, are found in the same LRLD event. These findings could explain some of the relationships found between LRLD regions in the bovine genome.

The number of LRLD events that involve genes showing interactions was much smaller than the total number of LRLD events in the three breeds. The existence of interactions between genes and genetic regulatory elements on distant genomic regions might also explain some of the observed LRLD events. Indeed, many studies have shown long-distance interactions between non-coding elements of the genome, especially with Hi-C data (e.g. [50–52]). However, since regulatory regions are not well annotated in the bovine genome, it is currently difficult to identify this type of interaction.

We checked, in the cattle QTL database, the presence of QTL associated with the same phenotype in each block of LRLD events. We found five and nine LRLD events showing QTL associated with the same phenotype in CHA and BLA, respectively. Only traits that are selected by breeders in beef cattle were checked, which could explain the small number of overlaps observed between QTL and LRLD events. Indeed, phenotypes related to dairy cattle have been much more widely studied and are thus over-represented in the database. Nevertheless, LRLD events may be an indicator of epistasis between several regions and thus may impact GWAS. Epistasis is considered as one potential explanation of the “missing heritability” [53, 54]. However, computational requirements are a challenge for the detection of epistatic interactions. Using the LRLD information in a GWAS model could help to reduce the complexity by testing the interactions between SNPs in LRLD blocks.

Another possible explanation for some of the detected LRLD events is the presence of errors in the genome assembly. The new bovine genome assembly (ARS-UCD1.2) used in this study has an increased assembly accuracy [55]. However, assembly errors, such as duplications or inversions, can still remain and create false positive LRLD events.

In this study, we identified LRLD events in beef cattle and also confirmed the inversely proportional relationship between LD and the physical distance between markers. These results are consistent with the literature. A study conducted by Hozé et al*.* [27] on 16 dairy and beef cattle breeds showed that the average LD drops to around 0.1 within a distance of 100 kb. Khatkar et al*.* [10] estimated that LD values drop to less than 0.08 for distances between markers greater than 200 kb in the Australian Holstein–Friesian cattle. The same result was reported by McKay et al*.* [17] in a study on eight cattle populations, in which the extent of LD did not exceed 500 kb. Beghain et al*.* [11] reported useful LD values ($r^{2}$ > 0.2) up to 724 kb in a separate population of BLA animals and since family structure has been observed in this population, it could be the main source of LD observed at such a distance.

In the CHA breed, BTA14 showed a lower level of LD decay with physical distance than the other chromosomes. Several QTL associated with growth, carcass, meat quality and eating quality traits have been identified on this chromosome in cattle (e.g. [56]). Furthermore, Allais et al*.* [57] identified a QTL associated with tenderness score on BTA14 (at position 59.5 cM) in the French Charolaise breed. One possible explanation for this large extent of LD could be the presence of genes that have a selective advantage or neutral markers that segregate with QTL on this chromosome. A study based on sequence data detected some loci under selection on this chromosome [58] such as the *PLAG1* gene, which is associated with stature and carcass yield [37]. This gene was located in the largest haplotype block observed in the three breeds.

## Conclusions

We conducted a linkage disequilibrium study on three populations of French beef cattle breeds genotyped on HD chips and report, for the first time, long-range linkage disequilibrium in these three breeds. Several hypotheses can explain this observed long-range linkage disequilibrium, such as bottlenecks, population admixture and epistatic selection. Our results should be taken into account for future genome-wide association studies.

## Supplementary Information

**Additional file 1: Tables S1–S3.** Summary distribution and density of SNPs over the genome in the Charolaise (Table S1), Limousine (Table S2) and Blonde d’Aquitaine (Table S3) breeds. In each table are provided the number of SNPs per chromosome, the chromosome size (kb), average inter-distance (± SD) between markers (kb), and median and maximum distance between markers.

**Additional file 2: Figures S1–S3.** SNP density over autosomes. Each chromosome was divided in windows of 500 kb and SNP density was plotted. Summary distribution and density of SNPs over the genome in the Charolaise (Figure S1), Limousine (Figure S2) and Blonde d’Aquitaine (Figure S3) breeds. Each figure shows SNP density across the autosomes. Each chromosome is divided into windows of 500 kb and SNP density was plotted.

**Additional file 3: Figures S4, S5.** Family structure analysis in the Charolaise, Limousine and Blonde d’Aquitaine breeds using *SNPRelate R* package. **Figure S4.** Principal component analysis (PCA). **Figure S5.** Clustering analysis based on the matrix of genome-wide identity by state (IBS) pairwise distances.

**Additional file 4: Tables S4–S6.** Mean linkage disequilibrium (*r*^*2*^) according to physical distance ranges in 500-kb windows in Charolaise (Table S4), Limousine (Table S5) and Blonde d’Aquitaine (Table S6).

**Additional file 5: Tables S7–S9.** Mean linkage disequilibrium (*r*^2^) on each autosome (1–29) in 500-kb windows. Number of SNP pairs per chromosome, mean *r*^2^, standard deviation (sd), percent of SNP pairs with LD superior to 0.2 (% *r*^2^ > 0.2), and percent of SNP pairs with high LD (% *r*^2^ > 0.8) in Charolaise (Table S7), Limousine (Table S8) and Blonde d’Aquitaine (Table S9).

**Additional file 6: Figures S6–S8.** LD decay within physical distance on each autosome (1–29) in 500-kb windows in the Charolaise (Figure S6), Limousine (Figure S7) and Blonde d’Aquitaine breeds (Figure S8). Doted horizontal lines are the LD background (computed on a subset of non-syntenic SNPs).

**Additional file 7: Tables S10–S12.** Summary of haplotype blocks for the Charolais (Table S10), Limousine (Table S11) and Blonde d’Aquitaine (Table S12) breeds. Each table provides the number of SNPs (Nb SNPs) per chromosome, chromosome size, number of haplotype blocks per autosome (1–29), block coverage length (Mb), chromosome block coverage (in percent), number of SNPs in blocks (Nb SNPs in Blocks) and percent of SNPs in blocks.

**Additional file 8: Figure S9.** Distribution of haplotype block sizes on each autosome (1–29) for the Charolaise breed. The red dots show haplotype blocks with size ≥ 100 kb. Blue circles show regions with larger haplotypes.

**Additional file 9: Figure S10.** Distribution of haplotype block sizes on each autosome (1–29) for the Limousine breed. The red dots show haplotype blocks with size ≥ 100 kb. Blue circles show regions with larger haplotypes.

**Additional file 10: Figure S11.** Distribution of haplotype block sizes on each autosome (1–29) for the Blonde d’Aquitaine breed. The red dots show haplotype blocks with size ≥ 100 kb. Blue circles show regions with larger haplotypes.

**Additional file 11: Tables S13–S15.** List of large haplotype blocks in the Charolaise (Table S13), Limousine (Table S14) and Blonde d’Aquitaine (Table S15) breeds.

**Additional file 12: Tables S16–S18.** List of LRLD events identified in the Limousine (Table S16), Charolaise (Table S17) and Blonde d’Aquitaine (Table S18) breeds. Each table show the *r*^2^ threshold, the pair of blocks number (LRLD number), breed, chromosome (chr), start position of block 1 (start_block1), end position of block 1 (stop_block1), start position of block 2 (start_block2), end position of block 2 (stop_block2) and distance between blocks.

**Additional file 13: Table S19.** List of common LRLD events between two or all the three breeds.

**Additional file 14: Figure S12.** Chromosome-wide LRLD on the Charolaise (CHA), Limousine (LIM) and Blonde d’Aquitaine (BLA) autosomes. Green for CHA, Red for LIM and Blue for BLA. Plots were done using *Circos* software.

**Additional file 15: Tables S20–S22.** List of LRLD events with functional interactions in the Charolaise (Table S20), Limousine (Table S21) and Blonde d’Aquitaine (Table S22) breeds. STRINGdb shows the interacting proteins in each block in LRLD from the STRING database. Cattle QTL db shows the LRLD blocks with the same QTL name identified in the cattle QTL database.

## Data Availability

The data that support the findings of this study are available from the GEMBAL consortium but restrictions apply to the availability of these data, which were used under license for the current study, and so are not publicly available. Data are however available from the authors upon reasonable request and with permission of the GEMBAL consortium.
