# Supplementary material for: Long-range linkage disequilibrium in French beef cattle breeds
Source: Genet Sel Evol. 2021 Jul 23;53:63. doi: 10.1186/s12711-021-00657-8 (PMC8306006; doi:10.1186/s12711-021-00657-8)
Supplement: Supplementary file 1 — Additional file 1: Tables S1–S3. Summary distribution and density of SNPs over the genome in the Charolaise (Table S1), Limousine (Table S2) and Blonde d’Aquitaine (Table S3) breeds. In each table are provided the number of SNPs per chromosome, the chromosome size (kb), average inter-distance (± SD) between markers (kb), and median and maximum distance between markers. [file 12711_2021_657_MOESM1_ESM.docx]

**Additional file 1 Table S1: Summary distribution of SNPs over the genome in the Charolais (CHA) breed.**

| **BTA** | **Number of SNPs** | **Chromosome size (kb)** | **Average inter-distance (± SD) in kb** | **Median distance (kb)** | **Maximum distance (kb)** |
| --- | --- | --- | --- | --- | --- |
| 1 | 35077 | 157777.043 | 4.499 ±5.976 | 2.719 | 209.882 |
| 2 | 30108 | 136010.41 | 4.519 ±5.555 | 2.776 | 118.119 |
| 3 | 27094 | 120905.213 | 4.464 ±6.176 | 2.7 | 249.511 |
| 4 | 26858 | 119487.929 | 4.45 ±5.831 | 2.699 | 228.662 |
| 5 | 25662 | 120015.93 | 4.678 ±7.532 | 2.759 | 298.857 |
| 6 | 27290 | 117656.843 | 4.313 ±5.856 | 2.641 | 407.767 |
| 7 | 24709 | 109903.029 | 4.449 ±6.772 | 2.656 | 356.395 |
| 8 | 21019 | 113184.481 | 5.386 ±13.616 | 3.033 | 1636.229 |
| 9 | 23509 | 104577.043 | 4.45 ±5.831 | 2.707 | 149.088 |
| 10 | 24061 | 103138.985 | 4.288 ±9.465 | 2.482 | 1124.131 |
| 11 | 25334 | 106845.472 | 4.219 ±5.36 | 2.452 | 142.564 |
| 12 | 19794 | 87116.461 | 4.402 ±8.622 | 2.647 | 568.43 |
| 13 | 15667 | 83062.919 | 5.303 ±8.672 | 3.088 | 681.171 |
| 14 | 15701 | 82313.218 | 5.244 ±9.751 | 3.005 | 657.439 |
| 15 | 19038 | 84925.408 | 4.462 ±7.541 | 2.571 | 483.864 |
| 16 | 18647 | 80746.516 | 4.332 ±6.303 | 2.562 | 285.721 |
| 17 | 17690 | 73130.277 | 4.135 ±5.268 | 2.523 | 233.675 |
| 18 | 15522 | 65581.987 | 4.226 ±6.949 | 2.353 | 235.838 |
| 19 | 15079 | 63374.392 | 4.204 ±5.939 | 2.45 | 259.626 |
| 20 | 17411 | 71672.911 | 4.118 ±5.223 | 2.385 | 112.941 |
| 21 | 16121 | 69771.381 | 4.329 ±6.902 | 2.542 | 310.364 |
| 22 | 15160 | 60608.972 | 3.999 ±4.874 | 2.35 | 77.897 |
| 23 | 12280 | 52450.916 | 4.273 ±8.932 | 2.569 | 740.909 |
| 24 | 14304 | 62195.427 | 4.349 ±5.593 | 2.636 | 134.724 |
| 25 | 10571 | 42206.424 | 3.994 ±4.417 | 2.592 | 84.825 |
| 26 | 12468 | 51854.503 | 4.16 ±5.857 | 2.368 | 249.423 |
| 27 | 10782 | 44336.935 | 4.114 ±8.297 | 2.435 | 669.558 |
| 28 | 10702 | 45506.805 | 4.254 ±5.481 | 2.402 | 112.091 |
| 29 | 11602 | 50560.969 | 4.359 ±6.757 | 2.534 | 341.215 |
| **Total** | **559260** | **2480918.8** | **4.4 *±*7.0** | **2.6** | **1636.2** |

**Additional file 1 Table S2 Summary distribution of SNPs over the genome in the Limousine (LIM) breed.**

| **BTA** | **Number of SNPs** | **Chromosome size (kb)** | **Average inter-distance (± SD) in kb** | **Median distance (kb)** | **Maximum distance (kb)** |
| --- | --- | --- | --- | --- | --- |
| 1 | 33815 | 157777.043 | 4.667 ±6.203 | 2.795 | 204.326 |
| 2 | 28104 | 135979.67 | 4.84 ±7.266 | 2.883 | 626.152 |
| 3 | 26398 | 120905.213 | 4.581 ±6.362 | 2.732 | 291.275 |
| 4 | 26167 | 119565.587 | 4.571 ±5.964 | 2.78 | 228.662 |
| 5 | 25376 | 120015.93 | 4.731 ±7.32 | 2.837 | 470.462 |
| 6 | 26664 | 117656.843 | 4.414 ±5.298 | 2.749 | 120.475 |
| 7 | 23553 | 109880.196 | 4.666 ±7.038 | 2.741 | 229.618 |
| 8 | 20432 | 113184.481 | 5.541 ±13.673 | 3.095 | 1636.723 |
| 9 | 22841 | 104577.043 | 4.58 ±5.904 | 2.779 | 150.57 |
| 10 | 23119 | 103210.417 | 4.466 ±10.803 | 2.539 | 1343.908 |
| 11 | 24711 | 106845.472 | 4.325 ±5.453 | 2.527 | 145.413 |
| 12 | 18975 | 87116.461 | 4.592 ±8.684 | 2.746 | 594.41 |
| 13 | 15659 | 83062.919 | 5.306 ±7.51 | 3.156 | 328.716 |
| 14 | 15617 | 82313.218 | 5.272 ±10.127 | 3.056 | 820.754 |
| 15 | 18406 | 84925.408 | 4.615 ±7.945 | 2.652 | 483.864 |
| 16 | 18029 | 80746.516 | 4.48 ±6.456 | 2.647 | 275.723 |
| 17 | 17008 | 73109.65 | 4.3 ±5.578 | 2.56 | 233.675 |
| 18 | 15090 | 65549.647 | 4.345 ±7.274 | 2.414 | 235.838 |
| 19 | 14704 | 63374.392 | 4.311 ±5.843 | 2.56 | 172.137 |
| 20 | 16845 | 71672.911 | 4.256 ±5.481 | 2.457 | 150.346 |
| 21 | 15222 | 69778.868 | 4.585 ±7.899 | 2.655 | 310.364 |
| 22 | 14563 | 60608.972 | 4.163 ±5.112 | 2.465 | 77.897 |
| 23 | 11942 | 52450.916 | 4.394 ±8.6 | 2.63 | 668.513 |
| 24 | 14087 | 62195.427 | 4.416 ±5.732 | 2.698 | 134.724 |
| 25 | 10101 | 42210.11 | 4.18 ±4.727 | 2.696 | 84.825 |
| 26 | 12003 | 51854.503 | 4.321 ±6.109 | 2.446 | 219.837 |
| 27 | 10478 | 44336.935 | 4.233 ±8.825 | 2.524 | 669.558 |
| 28 | 10252 | 45398.711 | 4.43 ±5.755 | 2.488 | 76.751 |
| 29 | 11158 | 50723.299 | 4.547 ±7.789 | 2.558 | 368.154 |
| **Total** | **541319** | **2481026.76** | **4.56 *±*7.34** | **2.7** | **1636.7** |

**Additional file 3 Table S3: Summary distribution of SNPs over the genome in the Blonde Aquitaine (BLA) breed.**

| **BTA** | **Number of SNPs** | **Chromosome size (kb)** | **Average inter-distance (± SD) in kb** | **Median distance (kb)** | **Maximum distance (kb)** |
| --- | --- | --- | --- | --- | --- |
| 1 | 35160 | 157779.793 | 4.489 *±*5*.*959 | 2.702 | 204.326 |
| 2 | 29650 | 135983.935 | 4.587 *±*6*.*79 | 2.745 | 575.689 |
| 3 | 27298 | 120918.029 | 4.431 *±*6*.*1 | 2.658 | 196.151 |
| 4 | 27100 | 119487.929 | 4.41 *±*5*.*744 | 2.694 | 228.662 |
| 5 | 26212 | 120015.93 | 4.58 *±*6*.*91 | 2.715 | 298.857 |
| 6 | 28138 | 117656.843 | 4.183 *±*4*.*977 | 2.608 | 132.072 |
| 7 | 24898 | 109903.029 | 4.415 *±*6*.*535 | 2.623 | 247.963 |
| 8 | 21095 | 113184.481 | 5.367 *±*13*.*36 | 3.036 | 1636.229 |
| 9 | 23727 | 104577.043 | 4.409 *±*5*.*707 | 2.706 | 149.088 |
| 10 | 24237 | 103138.985 | 4.257 *±*9*.*142 | 2.449 | 1073.239 |
| 11 | 25360 | 106845.472 | 4.214 *±*5*.*438 | 2.432 | 147.028 |
| 12 | 19637 | 87116.461 | 4.438 *±*8*.*743 | 2.643 | 594.41 |
| 13 | 15849 | 83067.28 | 5.242 *±*7*.*117 | 3.085 | 284.617 |
| 14 | 16409 | 82313.218 | 5.018 *±*7*.*41 | 2.963 | 456.869 |
| 15 | 19289 | 84925.408 | 4.404 *±*7*.*41 | 2.542 | 483.864 |
| 16 | 19015 | 80746.516 | 4.248 *±*5*.*449 | 2.554 | 130.082 |
| 17 | 17568 | 73130.277 | 4.164 *±*5*.*465 | 2.497 | 246.927 |
| 18 | 15700 | 65549.647 | 4.176 *±*6*.*915 | 2.3 | 219.605 |
| 19 | 15349 | 63374.392 | 4.13 *±*5*.*725 | 2.434 | 172.137 |
| 20 | 17389 | 71718.066 | 4.126 *±*5*.*338 | 2.384 | 112.941 |
| 21 | 16181 | 69771.381 | 4.313 *±*6*.*726 | 2.554 | 254.718 |
| 22 | 15003 | 60608.972 | 4.041 *±*5*.*038 | 2.348 | 100.851 |
| 23 | 12350 | 52450.916 | 4.248 *±*8*.*529 | 2.553 | 668.513 |
| 24 | 14516 | 62195.427 | 4.286 *±*5*.*674 | 2.588 | 134.724 |
| 25 | 10675 | 42210.11 | 3.955 *±*4*.*481 | 2.55 | 84.825 |
| 26 | 12580 | 51854.503 | 4.123 *±*5*.*707 | 2.332 | 219.837 |
| 27 | 10880 | 44336.935 | 4.076 *±*8*.*59 | 2.435 | 669.558 |
| 28 | 10672 | 45414.188 | 4.257 *±*5*.*555 | 2.402 | 112.091 |
| 29 | 11803 | 50723.299 | 4.299 *±*6*.*74 | 2.478 | 341.215 |
| **Total** | **563740** | **2480998.465** | **4.4 *±*6*.*9** | **2.6** | **1636.2** |
