# Supplementary material for: Long-range linkage disequilibrium in French beef cattle breeds
Source: Genet Sel Evol. 2021 Jul 23;53:63. doi: 10.1186/s12711-021-00657-8 (PMC8306006; doi:10.1186/s12711-021-00657-8)

**Additional file 2 Figure S1: SNP density over CHA autosomes (1-29).**


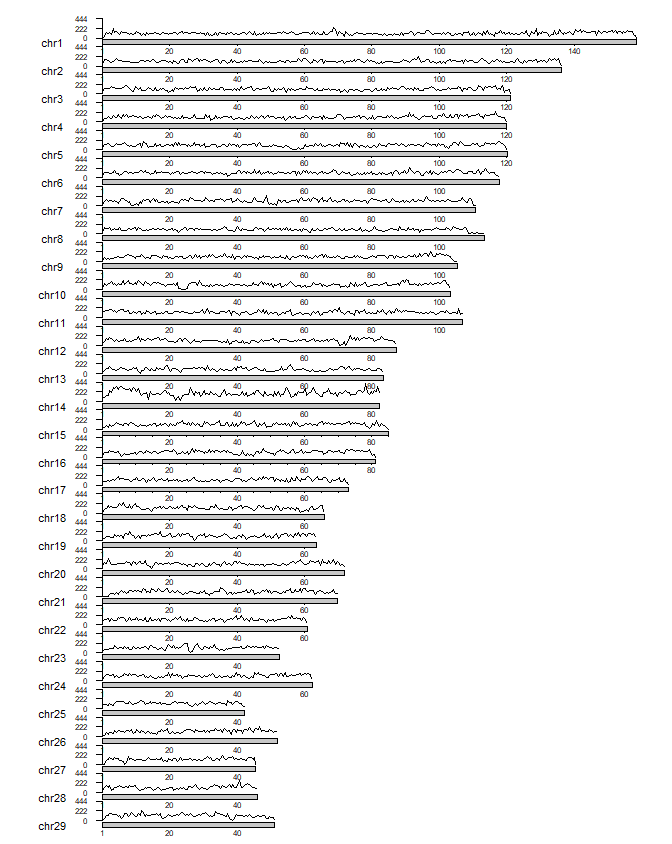


**Additional file 2 Figure S2: SNP density over LIM autosomes (1-29).**


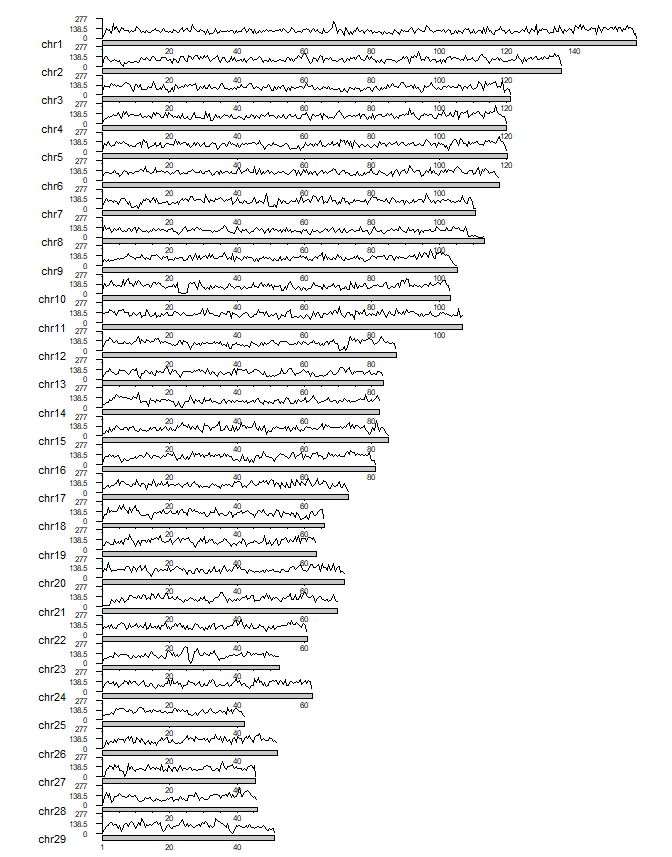


**Additional file 2 Figure S3: SNP density over BLA autosomes (1-29).**
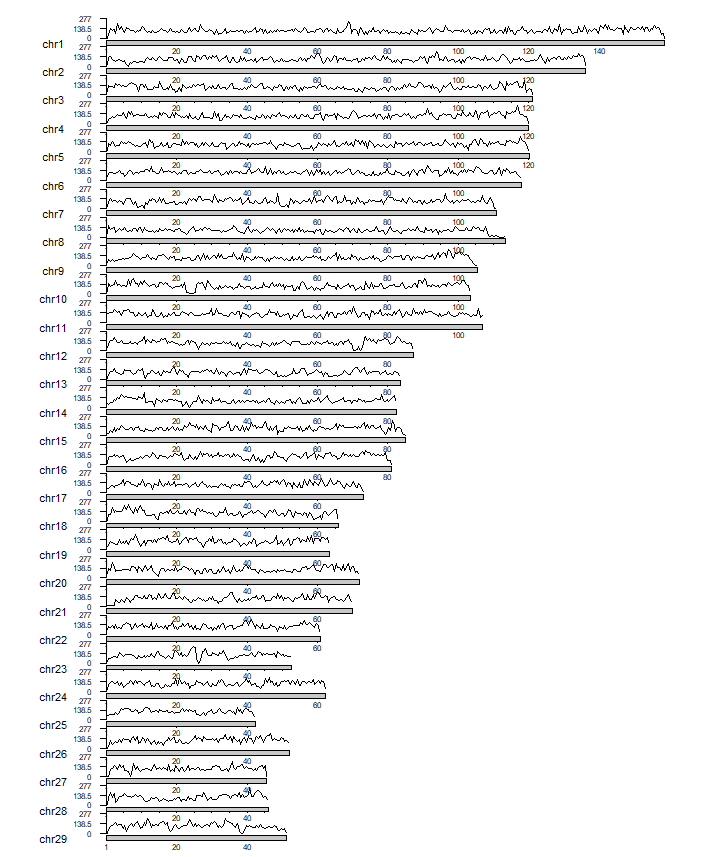

Supplement: Supplementary file 2 — Additional file 2: Figures S1–S3. SNP density over autosomes. Each chromosome was divided in windows of 500 kb and SNP density was plotted. Summary distribution and density of SNPs over the genome in the Charolaise (Figure S1), Limousine (Figure S2) and Blonde d’Aquitaine (Figure S3) breeds. Each figure shows SNP density across the autosomes. Each chromosome is divided into windows of 500 kb and SNP density was plotted. [file 12711_2021_657_MOESM2_ESM.docx]
