# Supplementary material for: Long-range linkage disequilibrium in French beef cattle breeds
Source: Genet Sel Evol. 2021 Jul 23;53:63. doi: 10.1186/s12711-021-00657-8 (PMC8306006; doi:10.1186/s12711-021-00657-8)
Supplement: Supplementary file 3 — Additional file 3: Figures S4, S5. Family structure analysis in the Charolaise, Limousine and Blonde d’Aquitaine breeds using SNPRelate R package. Figure S4. Principal component analysis (PCA). Figure S5. Clustering analysis based on the matrix of genome-wide identity by state (IBS) pairwise distances. [file 12711_2021_657_MOESM3_ESM.pdf]

Additional file 3: Family structure analysis in the CHA, LIM and BLA breeds using SNPRelate R package.

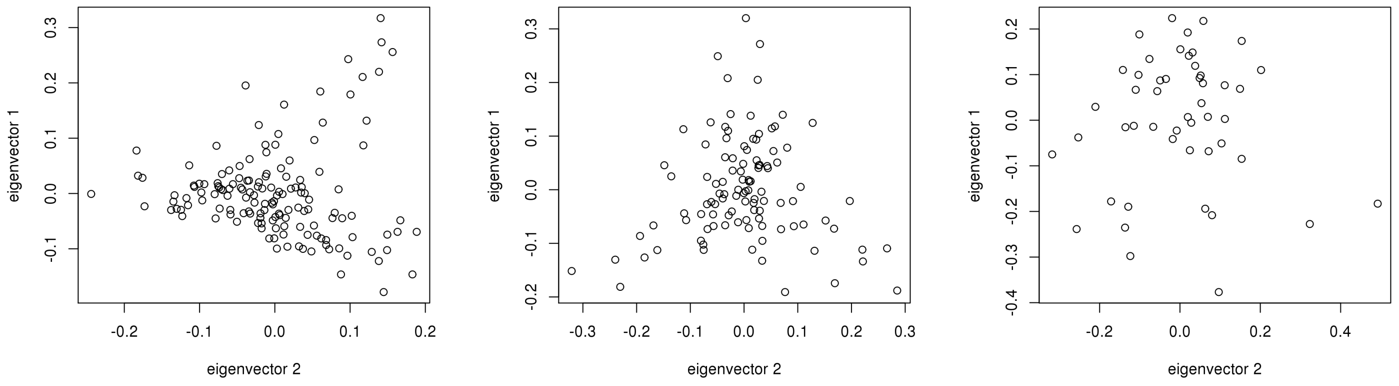

(a) Axis 1 explain 1.12 % of the total variance and axis 2 explain 1.01 %.

(b) Axis 1 explain 1.12 % of the total variance and axis 2 explain 1.01 %.

(c) Axis 1 explain 1.12 % of the total variance and axis 2 explain 1.01 %.

Figure S4: Principal component analysis (PCA) on the CHA (a), LIM (b) and BLA (c) populations.

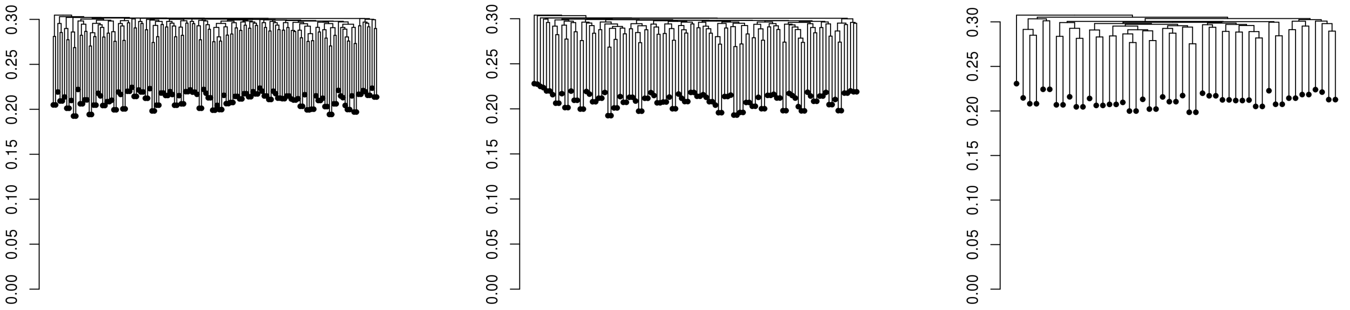

(a) (b) (c)

Figure S5: Cluster analysis plot on a matrix of genome-wide IBS pairwise distances for CHA (a), LIM (b) and BLA (c) individuals.
