# Supplementary material for: Long-range linkage disequilibrium in French beef cattle breeds
Source: Genet Sel Evol. 2021 Jul 23;53:63. doi: 10.1186/s12711-021-00657-8 (PMC8306006; doi:10.1186/s12711-021-00657-8)
Supplement: Supplementary file 6 — Additional file 6: Figures S6–S8. LD decay within physical distance on each autosome (1–29) in 500-kb windows in the Charolaise (Figure S6), Limousine (Figure S7) and Blonde d’Aquitaine breeds (Figure S8). Doted horizontal lines are the LD background (computed on a subset of non-syntenic SNPs). [file 12711_2021_657_MOESM6_ESM.pdf]

Additional file 6: LD decay within physical distance on each autosome (1-29) in a window of 500 kb. Doted horizontal lines are the LD background (computed on a subset of non-syntenic SNPs).

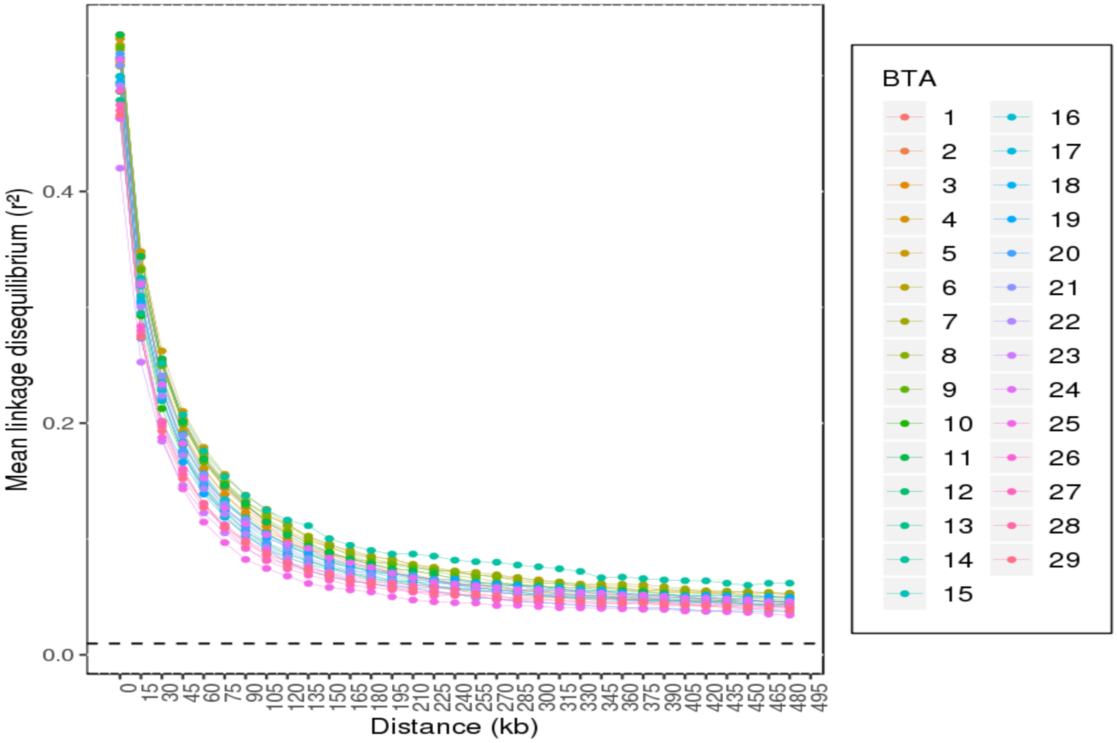

Figure S6: Distribution of average r^2 values for CHA breed with respect to physical distance (kb) in a window of 500 kb

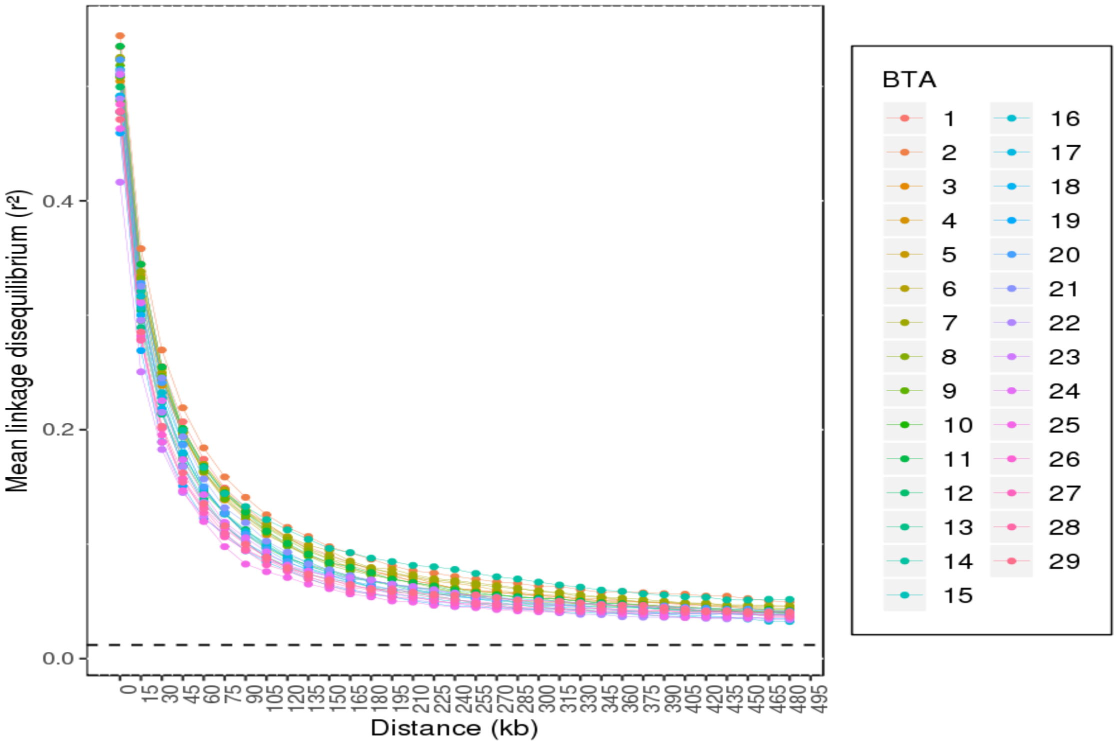

Figure S7: Distribution of average r^2 values for LIM breed with respect to physical distance (kb) in a window of 500 kb

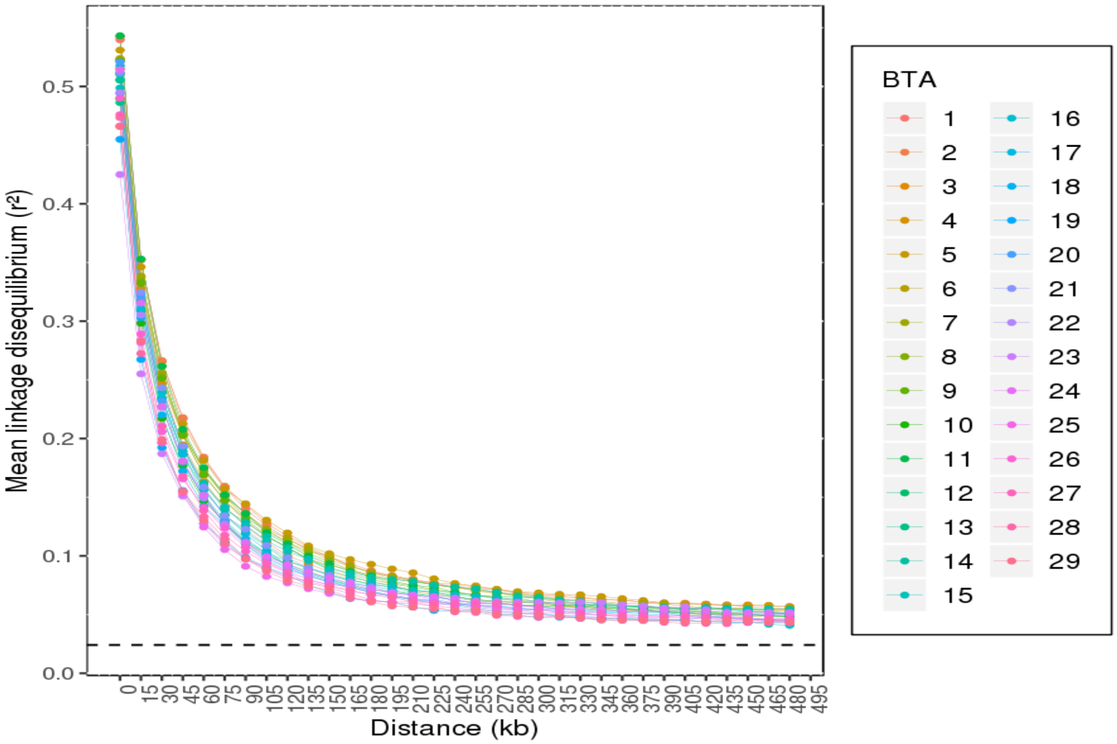

Figure S8: Distribution of average r^2 values for BLA breed with respect to physical distance (kb) in a window of 500 kb
