# Supplementary material for: Long-range linkage disequilibrium in French beef cattle breeds
Source: Genet Sel Evol. 2021 Jul 23;53:63. doi: 10.1186/s12711-021-00657-8 (PMC8306006; doi:10.1186/s12711-021-00657-8)
Supplement: Supplementary file 7 — Additional file 7: Tables S10–S12. Summary of haplotype blocks for the Charolais (Table S10), Limousine (Table S11) and Blonde d’Aquitaine (Table S12) breeds. Each table provides the number of SNPs (Nb SNPs) per chromosome, chromosome size, number of haplotype blocks per autosome (1–29), block coverage length (Mb), chromosome block coverage (in percent), number of SNPs in blocks (Nb SNPs in Blocks) and percent of SNPs in blocks. [file 12711_2021_657_MOESM7_ESM.pdf]

Table S10: Haplotype block summary for the CHA breed. Nb SNPs: Number of SNPs, Block cov: Block coverage, % SNPs in blocks: Number of SNPs in blocks (in percent)

BTA	Nb SNPs	Size (Mb)	Number of blocks	Block cov length (Mb)	Chr block cov (%)	Nb SNPs in Blocks	% SNPs in Blocks
1	35077	157.78	3166	102.14	64.74	29440	83.93
2	30108	136.01	2733	90.14	66.28	25486	84.65
3	27094	120.91	2521	78.78	65.16	22684	83.72
4	26858	119.49	2528	75.26	62.99	22372	83.30
5	25662	120.02	2352	78.46	65.37	21471	83.67
6	27290	117.66	2487	77.07	65.51	22980	84.21
7	24709	109.90	2243	74.10	67.42	20894	84.56
8	21019	113.18	1984	68.01	60.09	17149	81.59
9	23509	104.58	2159	68.53	65.53	19786	84.16
10	24061	103.14	2278	63.60	61.67	19544	81.23
11	25334	106.85	2275	70.13	65.63	21425	84.57
12	19794	87.12	1876	53.87	61.84	16269	82.19
13	15667	83.06	1587	45.60	54.90	12215	77.97
14	15701	82.31	1518	50.51	61.36	12771	81.34
15	19038	84.93	1782	53.63	63.15	15701	82.47
16	18647	80.75	1737	49.91	61.81	15339	82.26
17	17690	73.13	1670	44.24	60.49	14297	80.82
18	15522	65.58	1467	39.10	59.63	12650	81.50
19	15079	63.37	1488	37.25	58.77	11884	78.81
20	17411	71.67	1598	45.36	63.29	14482	83.18
21	16121	69.77	1504	45.22	64.81	13447	83.41
22	15160	60.61	1416	37.88	62.49	12338	81.39
23	12280	52.45	1296	27.61	52.65	9105	74.14
24	14304	62.20	1382	38.47	61.86	11786	82.40
25	10571	42.21	1106	25.42	60.23	8480	80.22
26	12468	51.85	1201	30.92	59.62	10057	80.66
27	10782	44.34	1081	25.49	57.50	8604	79.80
28	10702	45.51	1067	25.66	56.38	8376	78.27
29	11602	50.56	1162	29.41	58.17	9192	79.23
Total	559260	2480.92	52664	1551.78	62.55	460224	82.29

Table S11: Haplotype block summary for the LIM breed. Nb SNPs: Number of SNPs, Block cov: Block coverage, % SNPs in blocks: Number of SNPs in blocks (in percent)

BTA	Nb SNPs	Size (Mb)	Number of blocks	Block cov length (Mb)	Chr block cov (%)	Nb SNPs in Blocks	% SNPs in Blocks
1	33815	157.78	3058	99.50	63.06	27950	82.66
2	28104	135.98	2446	86.76	63.80	23187	82.50
3	26398	120.91	2427	76.81	63.53	21532	81.57
4	26167	119.57	2410	72.05	60.26	21065	80.50
5	25376	120.02	2328	73.50	61.24	20600	81.18
6	26664	117.66	2441	74.79	63.57	22022	82.59
7	23553	109.88	2110	69.64	63.38	19356	82.18
8	20432	113.18	1976	64.57	57.05	16206	79.32
9	22841	104.58	2067	64.70	61.87	18612	81.49
10	23119	103.21	2155	60.90	59.00	18371	79.46
11	24711	106.85	2173	67.66	63.32	20358	82.38
12	18975	87.12	1809	51.16	58.72	15099	79.57
13	15659	83.06	1545	44.58	53.67	11986	76.54
14	15617	82.31	1503	47.87	58.16	12322	78.90
15	18406	84.93	1733	51.48	60.62	14703	79.88
16	18029	80.75	1661	48.13	59.60	14489	80.36
17	17008	73.11	1613	43.08	58.92	13668	80.36
18	15090	65.55	1425	38.16	58.22	12024	79.68
19	14704	63.37	1437	36.02	56.84	11259	76.57
20	16845	71.67	1516	44.40	61.95	13773	81.76
21	15222	69.78	1446	42.60	61.05	12403	81.48
22	14563	60.61	1366	35.98	59.37	11451	78.63
23	11942	52.45	1263	26.92	51.33	8603	72.04
24	14087	62.20	1344	36.50	58.68	11299	80.21
25	10101	42.21	1019	24.06	57.01	7742	76.65
26	12003	51.85	1149	29.27	56.45	9312	77.58
27	10478	44.34	1033	24.10	54.36	8083	77.14
28	10252	45.40	1020	24.85	54.73	7858	76.65
29	11158	50.72	1080	28.24	55.68	8617	77.23
Total	541319	2481.03	50553	1488.29	59.99	433950	80.17

Table S12: Haplotype block summary for the BLA breed. Nb SNPs: Number of SNPs, Block cov: Block coverage, % SNPs in blocks: Number of SNPs in blocks (in percent)

BTA	Nb SNPs	Size (Mb)	Number of blocks	Block cov length (Mb)	Chr block cov (%)	Nb SNPs in Blocks	% SNPs in Blocks
1	35160	157.78	2925	91.28	57.85	27340	77.76
2	29650	135.98	2469	79.02	58.11	22959	77.43
3	27298	120.92	2346	68.53	56.67	20823	76.28
4	27100	119.49	2265	65.47	54.80	20330	75.02
5	26212	120.02	2216	68.16	56.79	20148	76.87
6	28138	117.66	2375	68.13	57.91	21788	77.43
7	24898	109.90	2059	63.71	57.97	19221	77.20
8	21095	113.18	1806	57.89	51.14	15395	72.98
9	23727	104.58	1986	59.72	57.11	18200	76.71
10	24237	103.14	2093	54.72	53.05	17986	74.21
11	25360	106.85	2074	61.88	57.92	19772	77.97
12	19637	87.12	1692	48.40	55.55	14820	75.47
13	15849	83.07	1412	38.89	46.82	11022	69.54
14	16409	82.31	1463	41.65	50.59	11887	72.44
15	19289	84.93	1634	46.08	54.25	14400	74.65
16	19015	80.75	1621	44.97	55.70	14392	75.69
17	17568	73.13	1527	38.66	52.86	13098	74.56
18	15700	65.55	1383	35.47	54.11	11778	75.02
19	15349	63.37	1347	31.93	50.39	10761	70.11
20	17389	71.72	1486	40.14	55.97	13434	77.26
21	16181	69.77	1408	39.43	56.51	12367	76.43
22	15003	60.61	1292	32.94	54.34	11129	74.18
23	12350	52.45	1104	24.06	45.87	8033	65.04
24	14516	62.20	1262	33.51	53.88	10930	75.30
25	10675	42.21	956	21.71	51.43	7628	71.46
26	12580	51.85	1101	26.77	51.63	9153	72.76
27	10880	44.34	977	21.94	49.49	7804	71.73
28	10672	45.41	965	22.10	48.66	7539	70.64
29	11803	50.72	1059	24.91	49.11	8332	70.59
Total	563740	2481.00	48303	1352.07	54.50	422469	74.94

Additional file 7: Haplotype blocks summary on the 3 breeds.
