# Supplementary material for: Long-range linkage disequilibrium in French beef cattle breeds
Source: Genet Sel Evol. 2021 Jul 23;53:63. doi: 10.1186/s12711-021-00657-8 (PMC8306006; doi:10.1186/s12711-021-00657-8)
Supplement: Supplementary file 9 — Additional file 9: Figure S10. Distribution of haplotype block sizes on each autosome (1–29) for the Limousine breed. The red dots show haplotype blocks with size ≥ 100 kb. Blue circles show regions with larger haplotypes. [file 12711_2021_657_MOESM9_ESM.pdf]

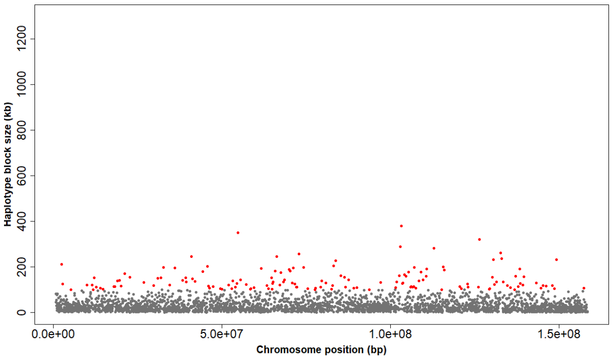

BTA1

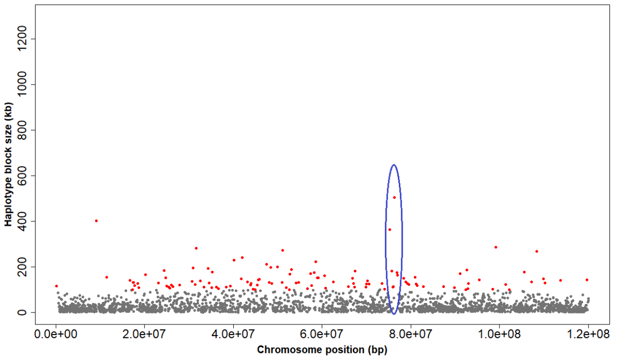

BTA5

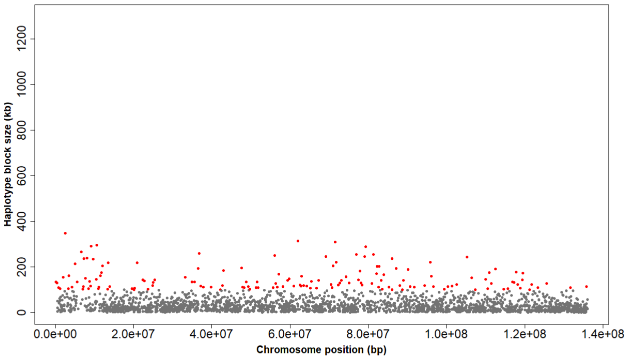

BTA2

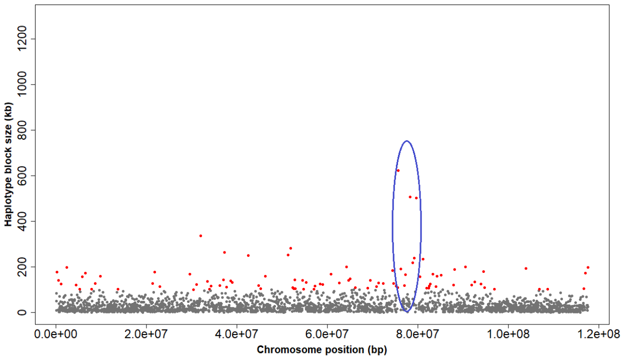

BTA6

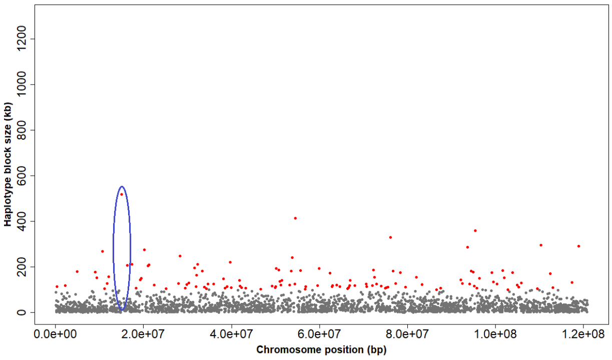

BTA3

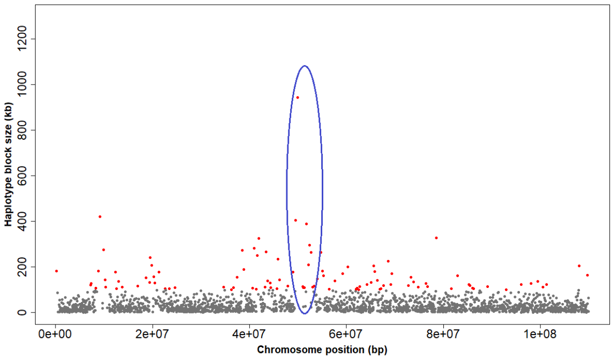

BTA7

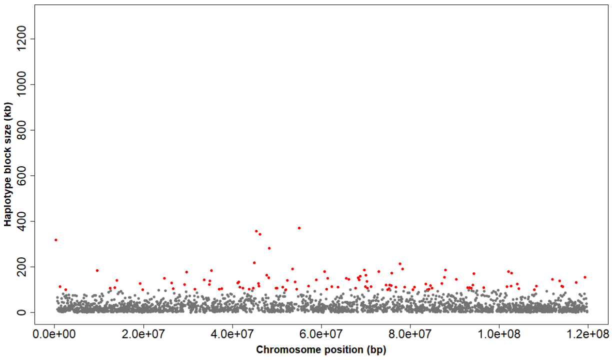

BTA4

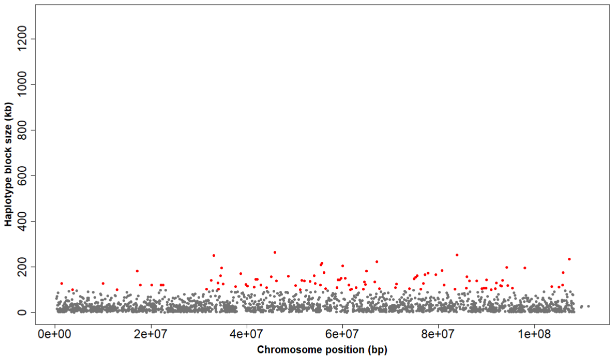

BTA8

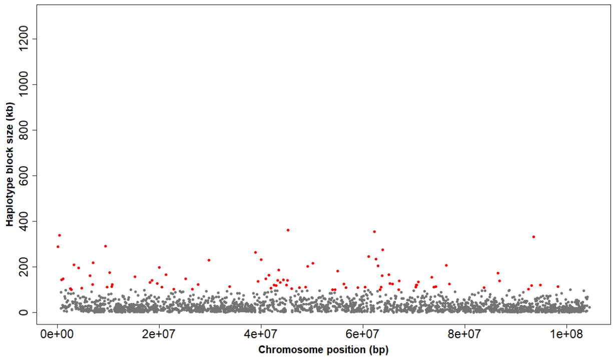

BTA9

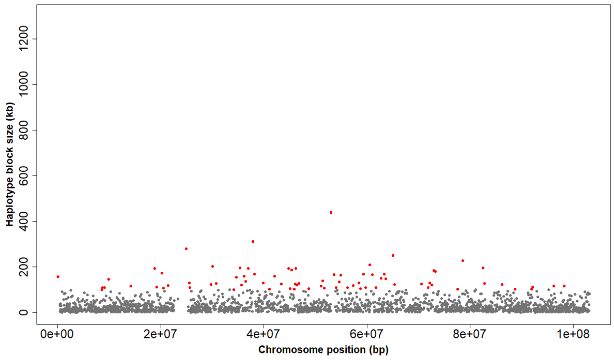

BTA10

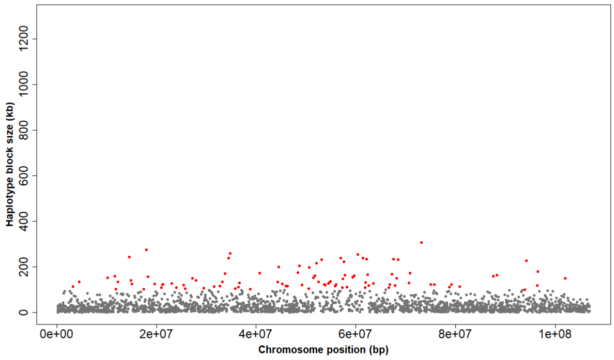

BTA11

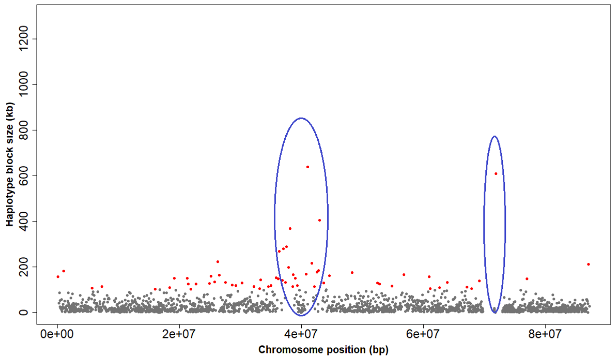

BTA12

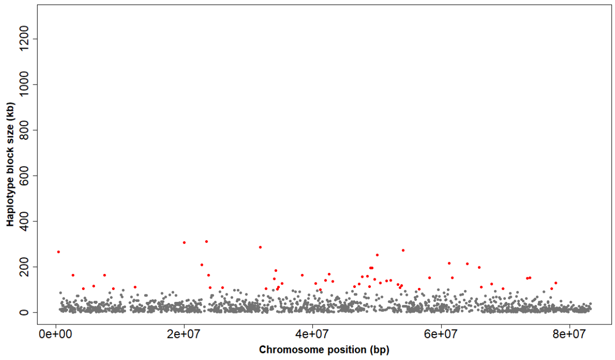

BTA13

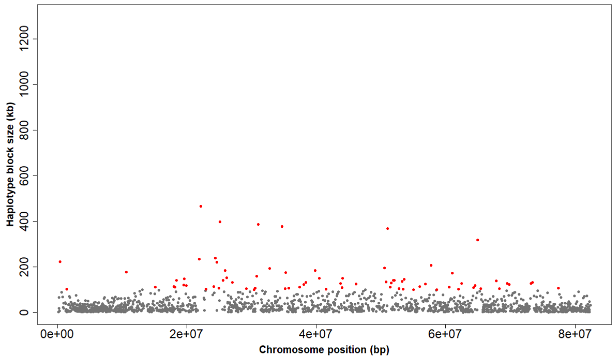

BTA14

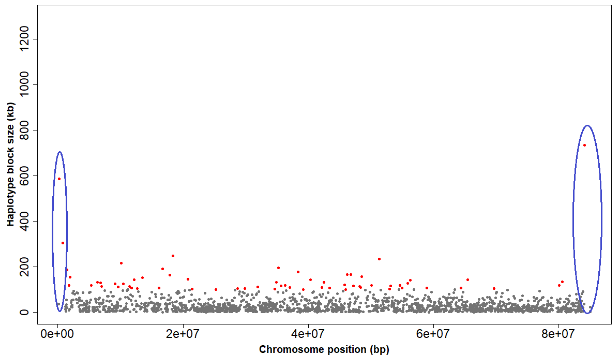

BTA15

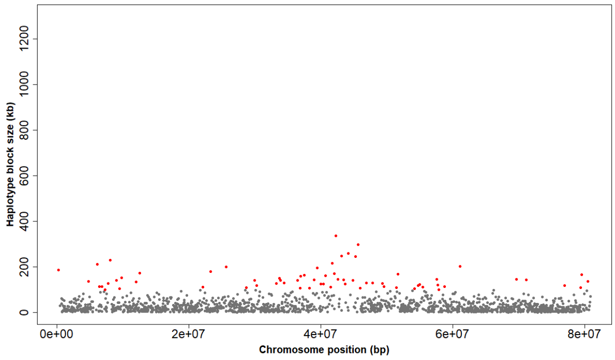

BTA16

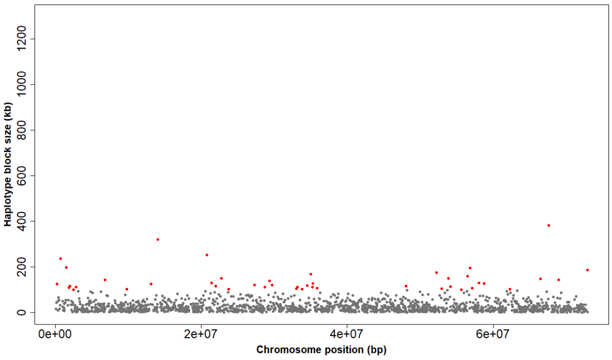

BTA17

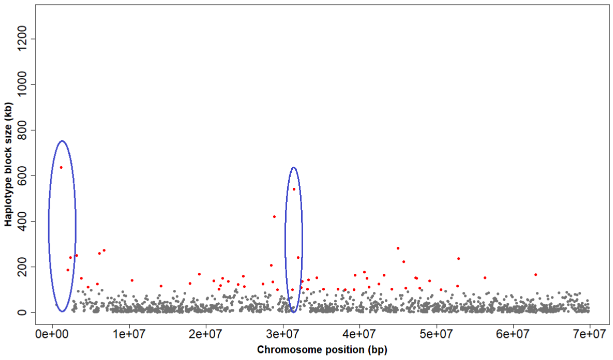

BTA21

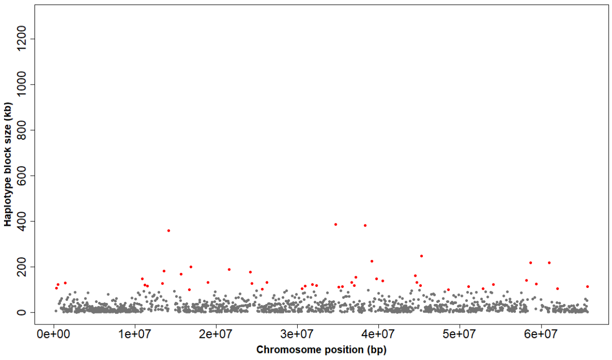

BTA18

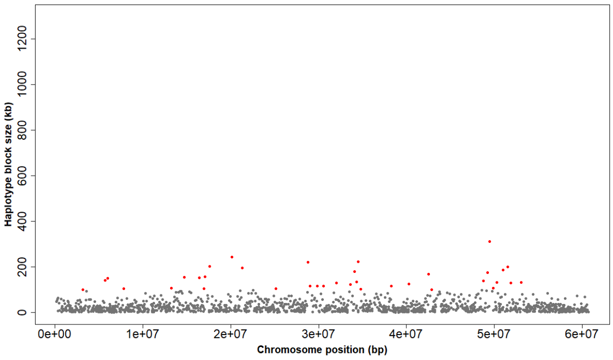

BTA22

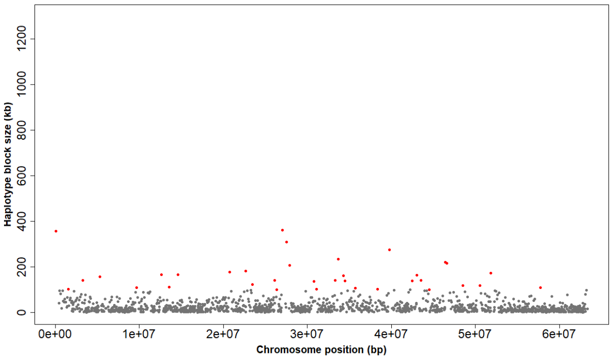

BTA19

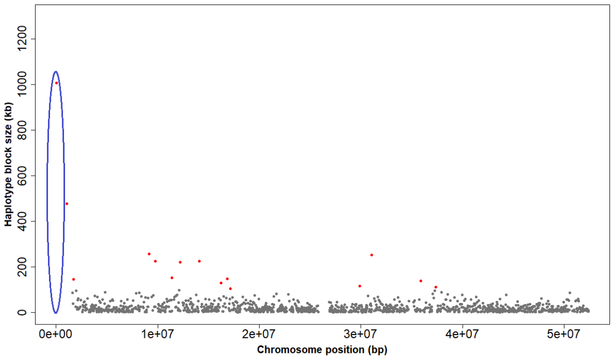

BTA23

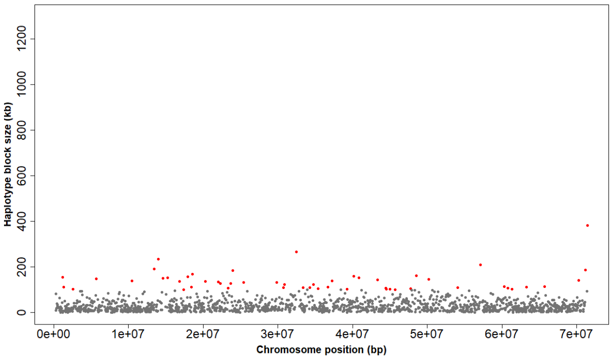

BTA20

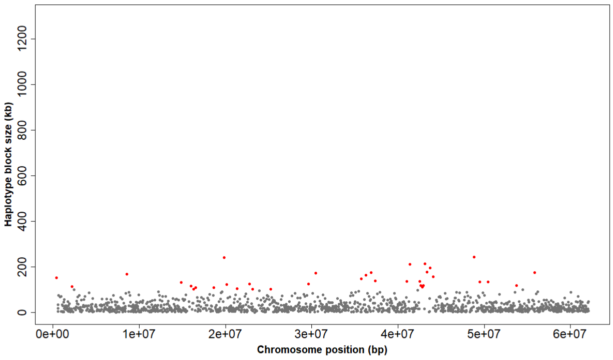

BTA24

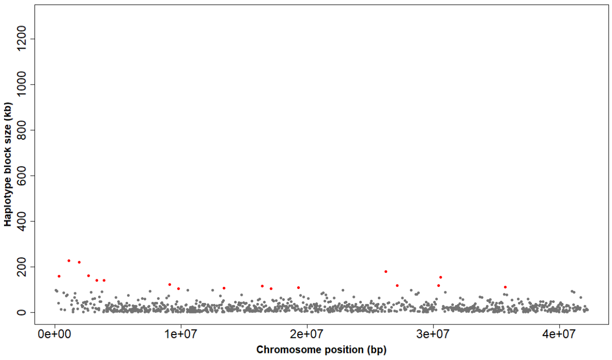

BTA25

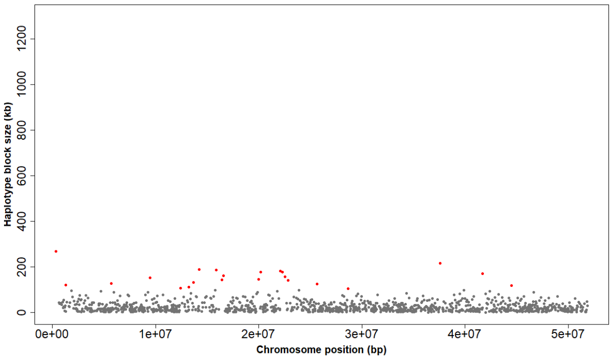

BTA26

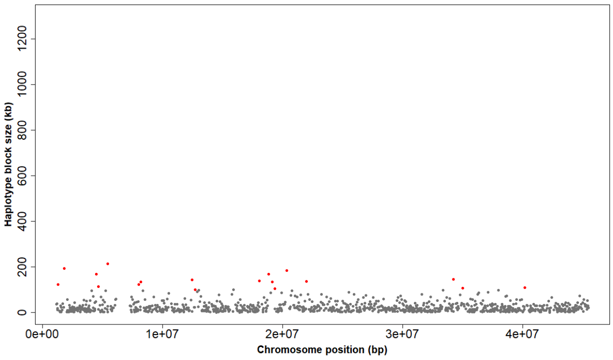

BTA27

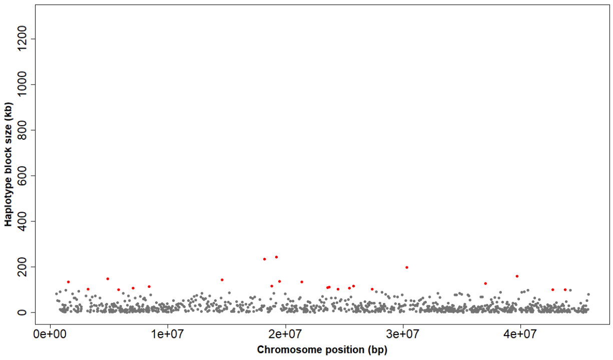

BTA28

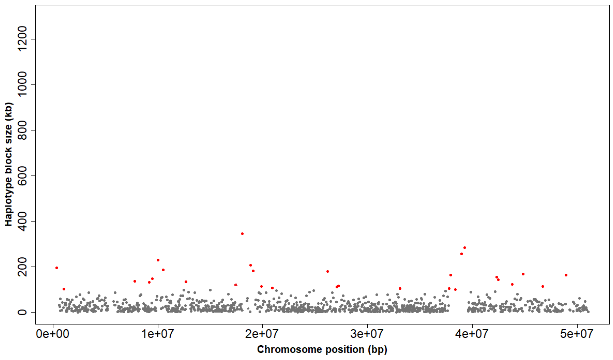

BTA29

Additional file 9:
Figure S10: Size and haplotype block position on the autosomes (1-29) of the Limousine (LIM) breed. Red points: haplotype blocks with size $>= 100$ kb. Blue circles: Area with larger haplotypes ($>=$ 500 kb)
