# Supplementary material for: Long-range linkage disequilibrium in French beef cattle breeds
Source: Genet Sel Evol. 2021 Jul 23;53:63. doi: 10.1186/s12711-021-00657-8 (PMC8306006; doi:10.1186/s12711-021-00657-8)
Supplement: Supplementary file 14 — Additional file 14: Figure S12. Chromosome-wide LRLD on the Charolaise (CHA), Limousine (LIM) and Blonde d’Aquitaine (BLA) autosomes. Green for CHA, Red for LIM and Blue for BLA. Plots were done using Circos software. [file 12711_2021_657_MOESM14_ESM.pdf]

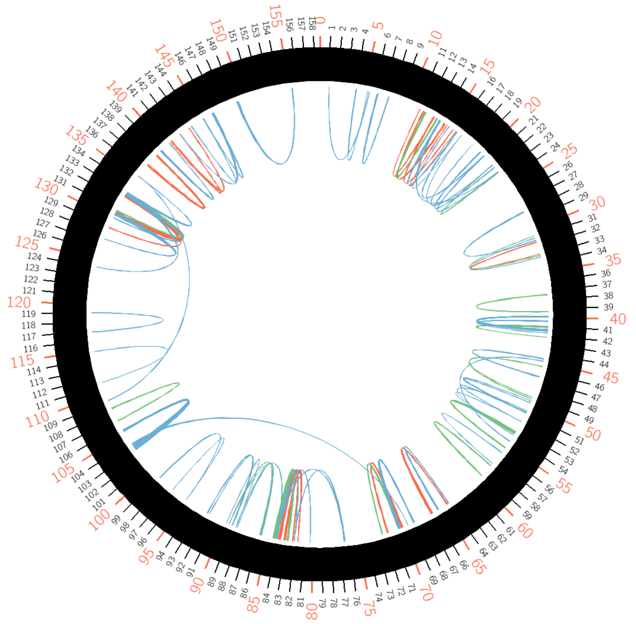

BTA1

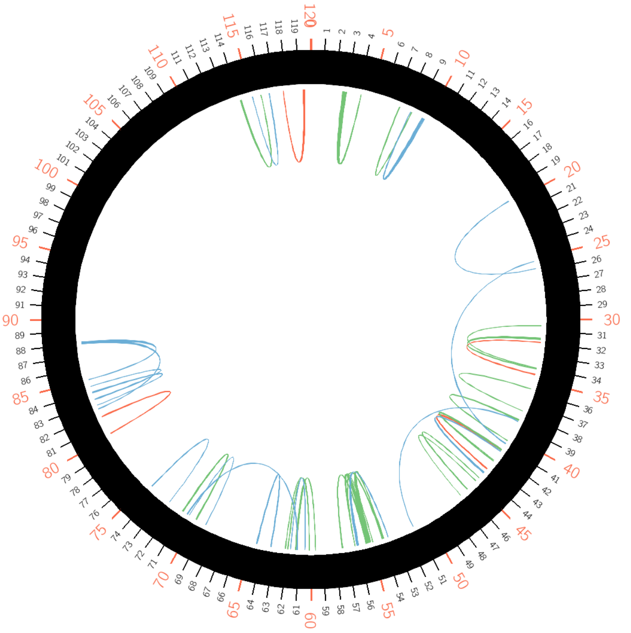

BTA4

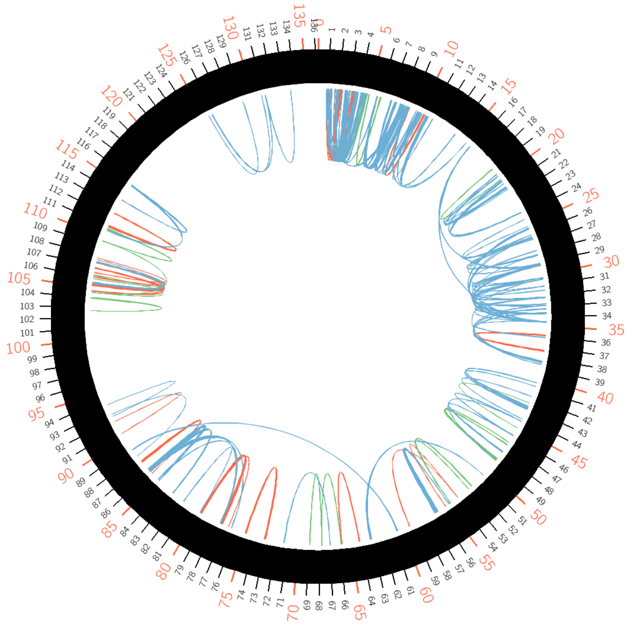

BTA2

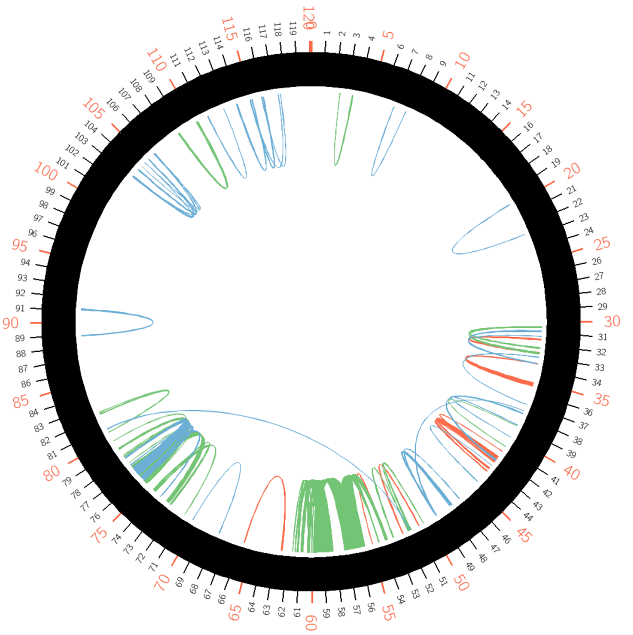

BTA5

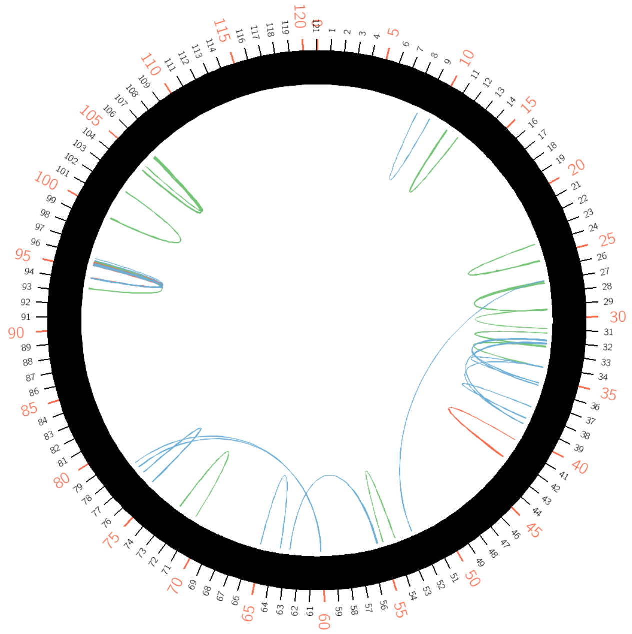

BTA3

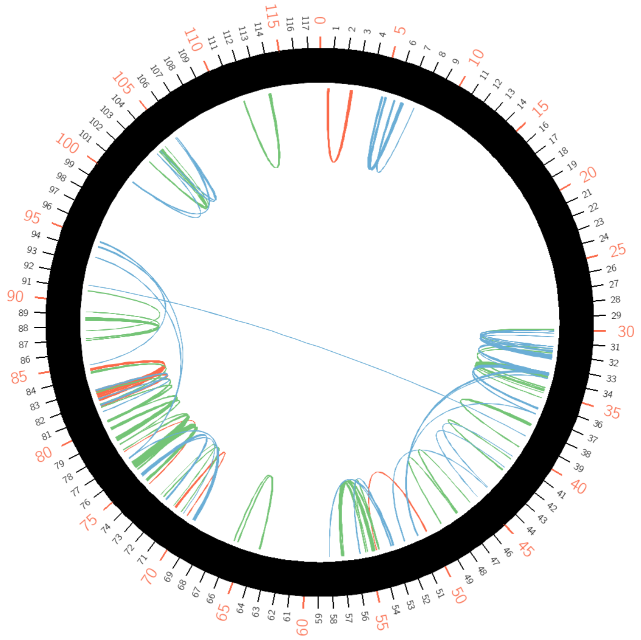

BTA6

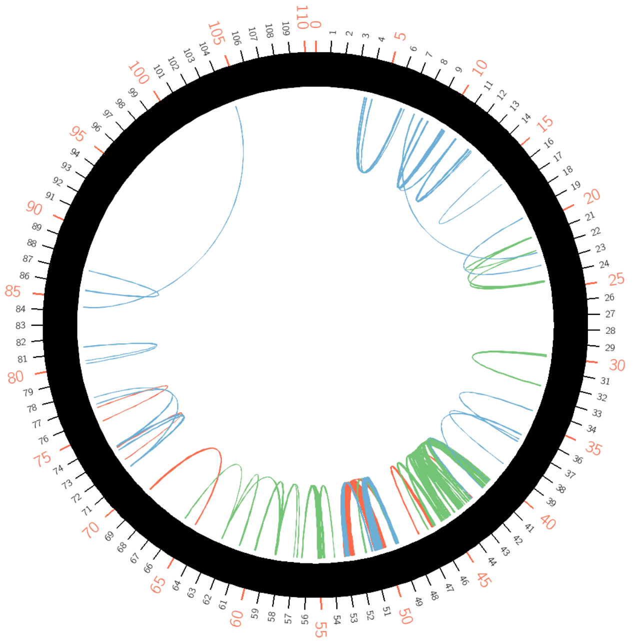

BTA7

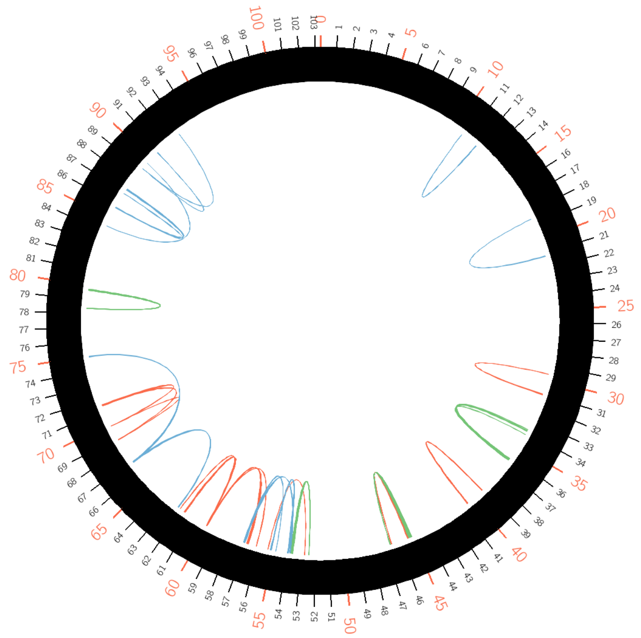

BTA10

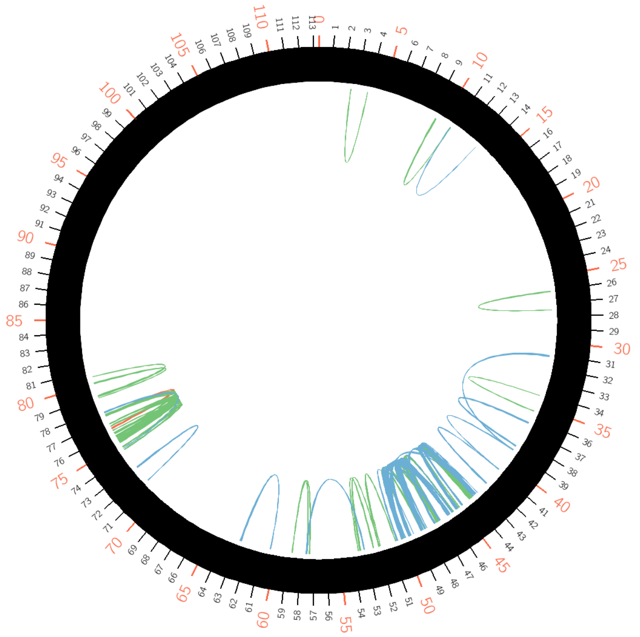

BTA8

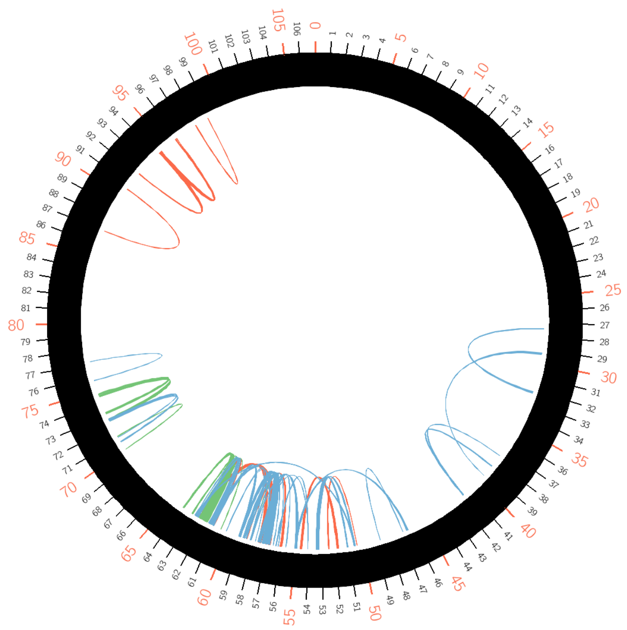

BTA11

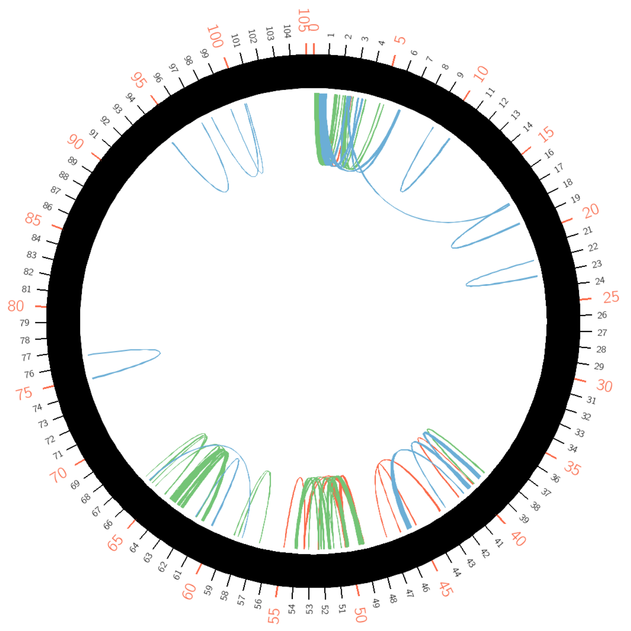

BTA9

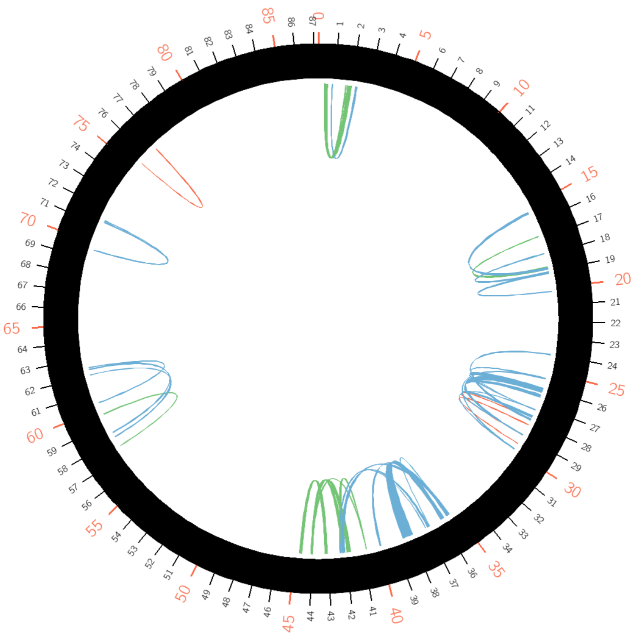

BTA12

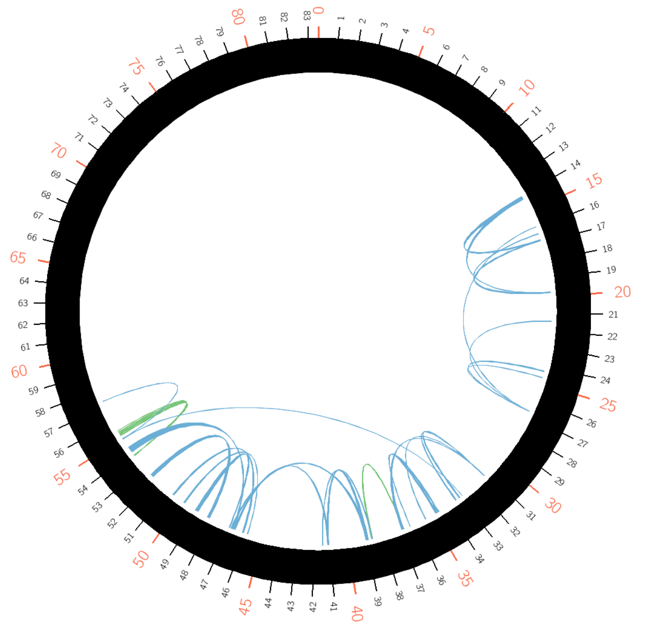

BTA13

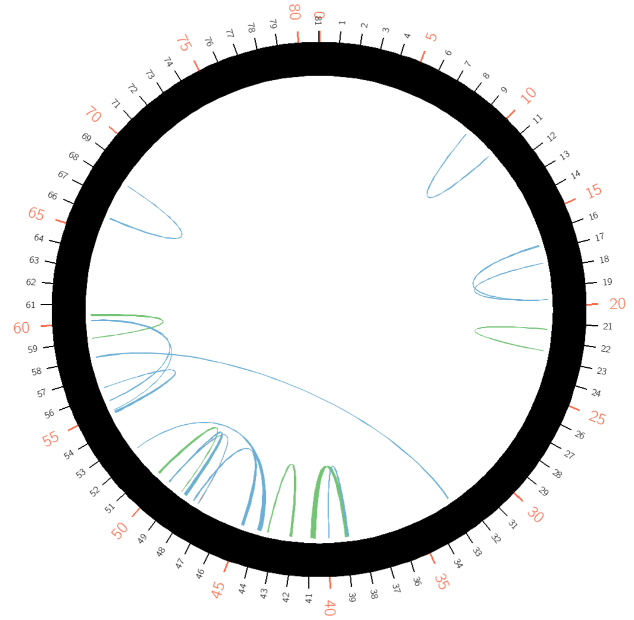

BTA16

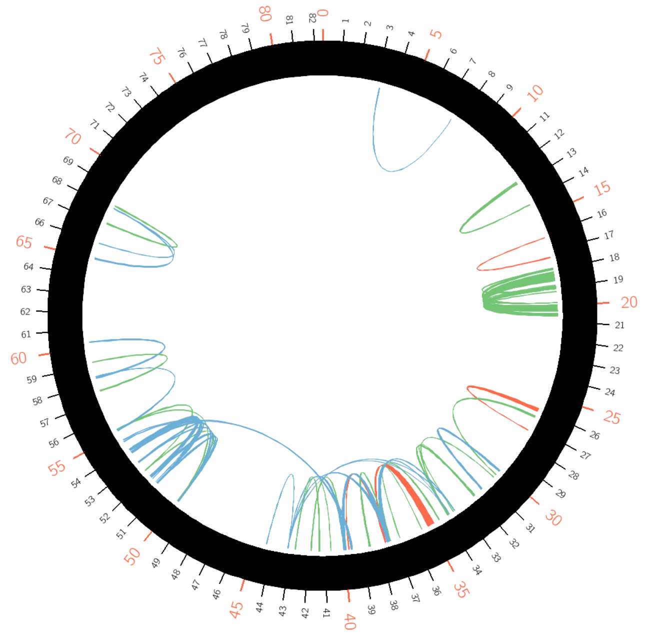

BTA14

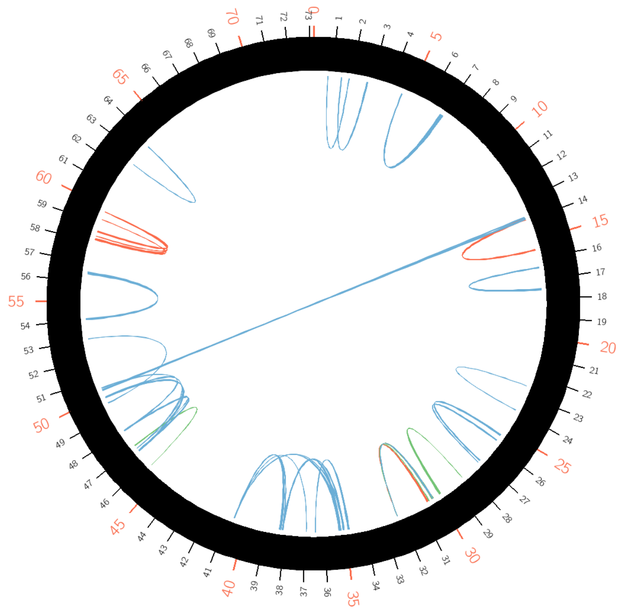

BTA17

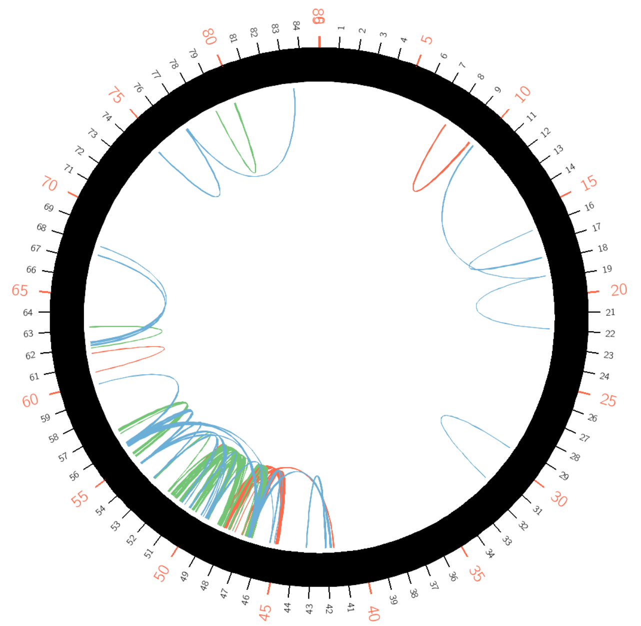

BTA15

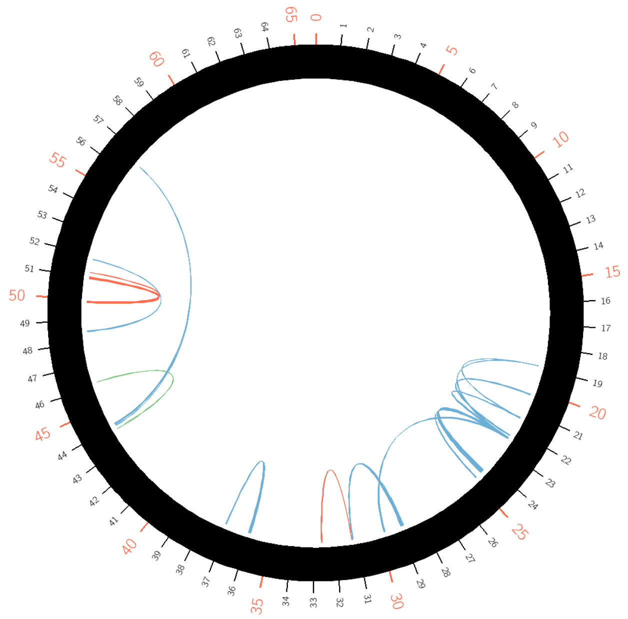

BTA18

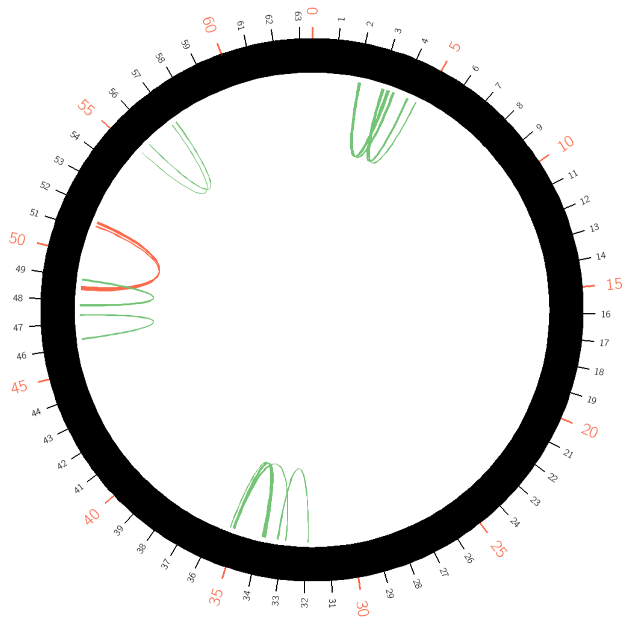

BTA19

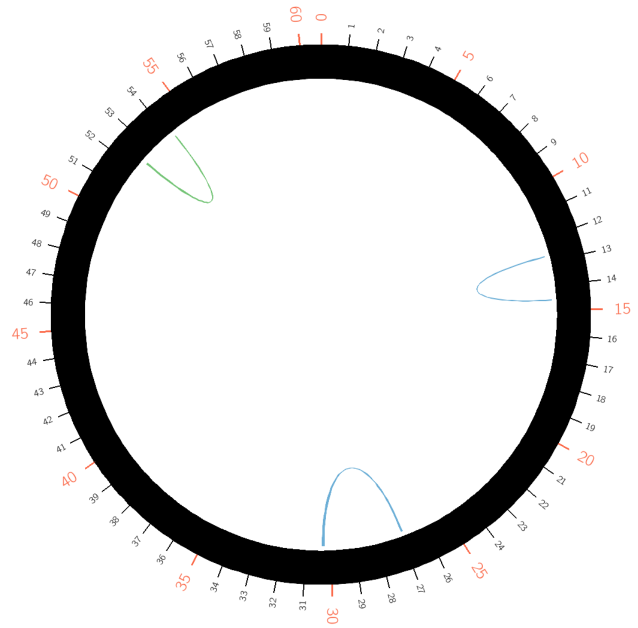

BTA22

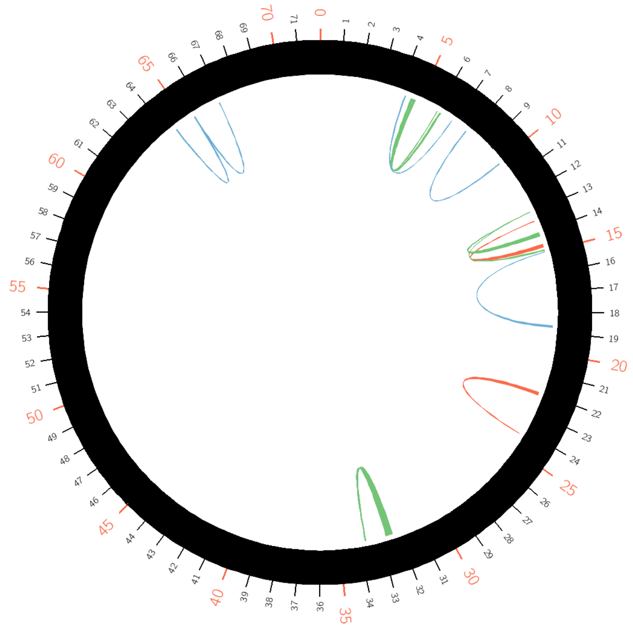

BTA20

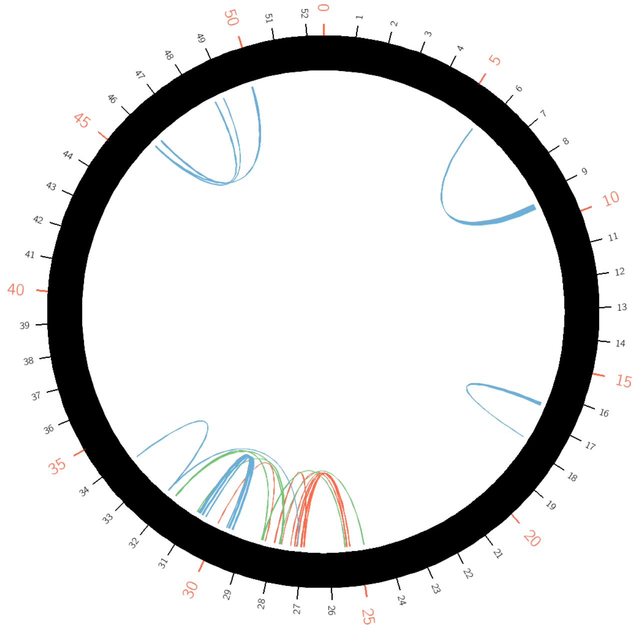

BTA23

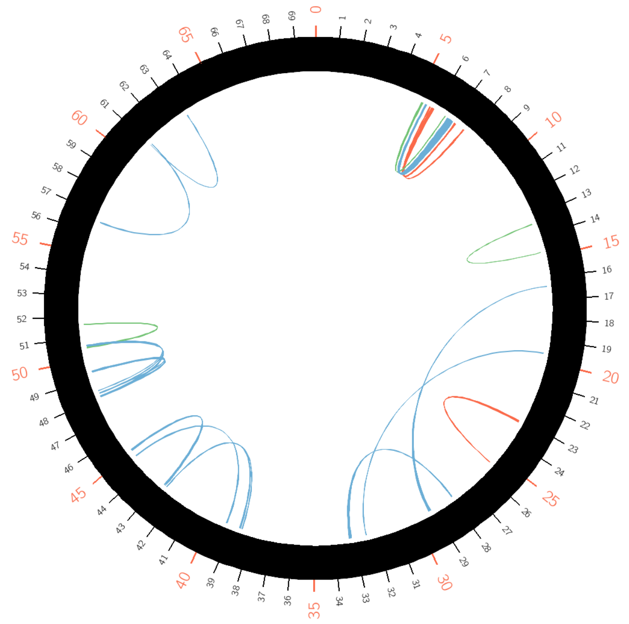

BTA21

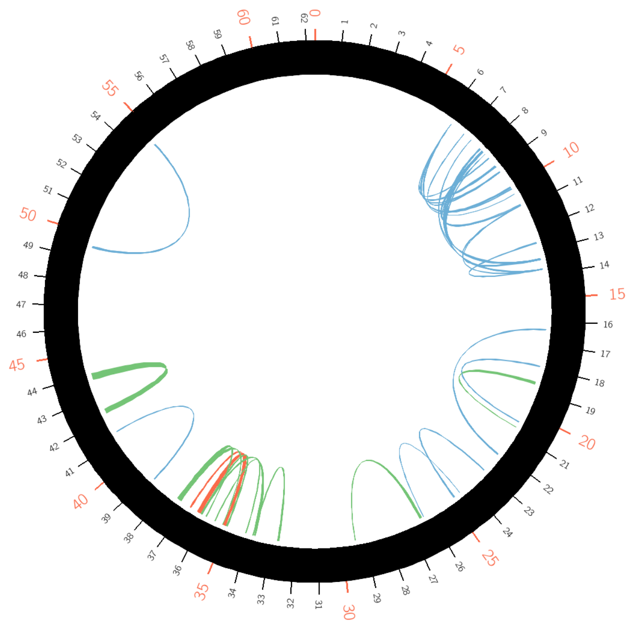

BTA24

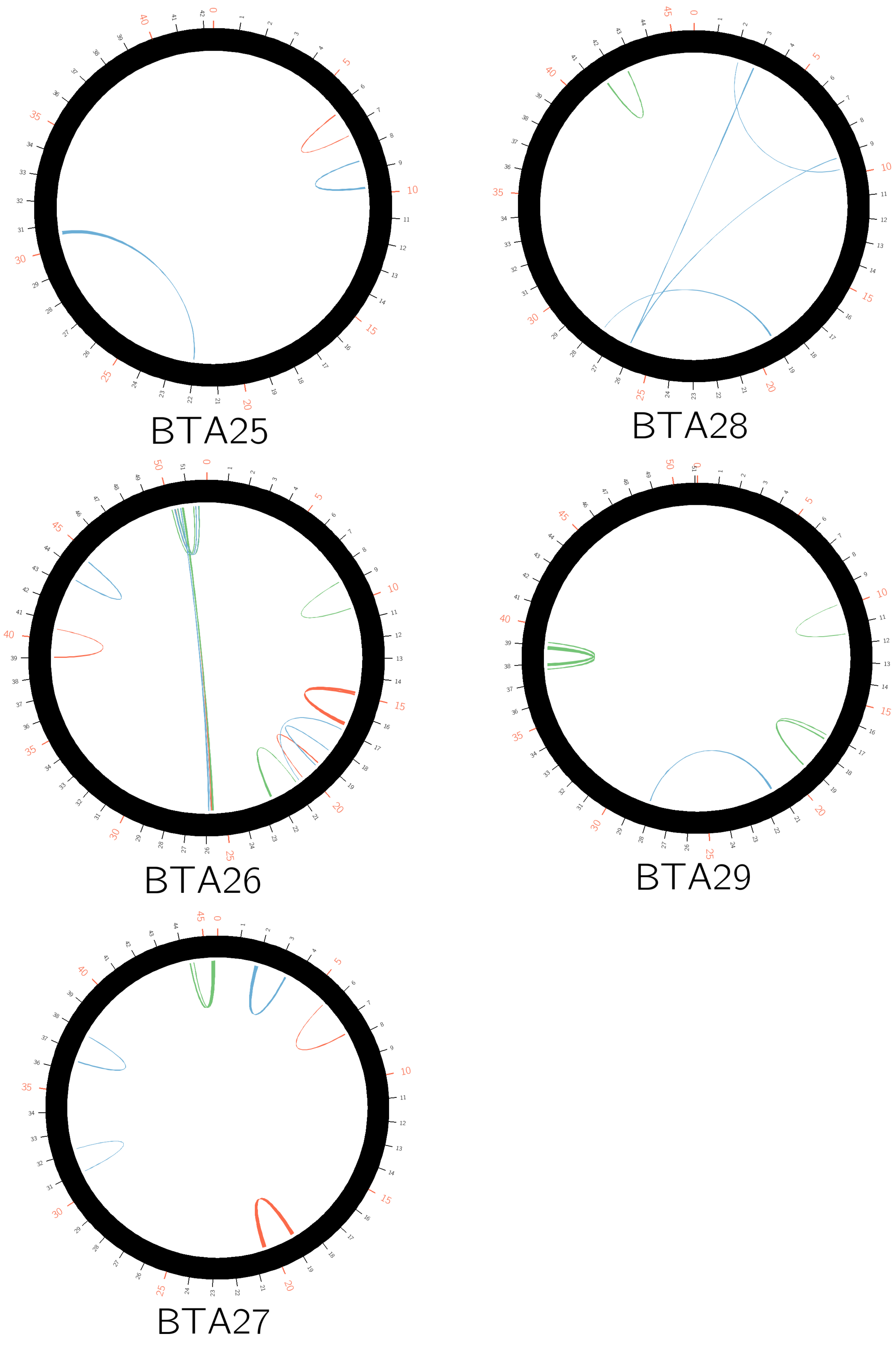

Additional file 14: Figure S12: Chromosome-wide LRLD on the CHA, LIM and BLA autosomes. Green for CHA, Red for LIM and Blue for BLA. Plots were done using circos software.
